# A population-based study on incidence trends of small intestine cancer in the United States from 2000 to 2020

**DOI:** 10.1371/journal.pone.0307019

**Published:** 2024-08-19

**Authors:** Seyed Ehsan Mousavi, Mehran Ilaghi, Vahid Mahdavizadeh, Rasoul Ebrahimi, Armin Aslani, Zahra Yekta, Seyed Aria Nejadghaderi

**Affiliations:** 1 Neurosciences Research Center, Aging Research Institute, Tabriz University of Medical Sciences, Tabriz, Iran; 2 Department of Community Medicine, Social Determinants of Health Research Center, Faculty of Medicine, Tabriz University of Medical Sciences, Tabriz, Iran; 3 Institute of Neuropharmacology, Kerman Neuroscience Research Center, Kerman University of Medical Sciences, Kerman, Iran; 4 Student Research Committee, Mashhad University of Medical Sciences, Mashhad, Iran; 5 School of Medicine, Shahid Beheshti University of Medical Sciences, Tehran, Iran; 6 Calaveras County Department of Health, Calaveras County, California, United States of America; 7 HIV/STI Surveillance Research Center, and WHO Collaborating Center for HIV Surveillance, Institute for Futures Studies in Health, Kerman University of Medical Sciences, Kerman, Iran; 8 Systematic Review and Meta‑analysis Expert Group (SRMEG), Universal Scientific Education and Research Network (USERN), Tehran, Iran; Universita degli Studi della Campania Luigi Vanvitelli, ITALY

## Abstract

**Background:**

Although rare, small intestine cancer is on the rise in the developed world. We aimed to investigate the incidence trends of small intestine cancer by sex, race/ethnicity, age, and histological subgroups in the United States (US) over 2000–2020. Also, we evaluated the COVID-19 impacts on the incidence trends of this cancer.

**Methods:**

Data were collected from the Surveillance, Epidemiology, and End Results 22 database. Both the average annual percent change (AAPC) and age-standardized incidence rates (ASIRs) were determined. The findings were expressed as counts and incidence rates adjusted for age per 100,000 people with 95% confidence intervals (CIs).

**Results:**

A total of 67,815 cases of small intestine cancer across all age groups were reported in the US between 2000 and 2019. Neuroendocrine carcinoma was the most often reported subtype (54.26%). The age group of 55 to 69 years (38.08%), men (53.10%), and Non-Hispanic Whites (69.07%) accounted for the majority of cases. Over 2000–2019, the ASIRs for small intestine cancer among men and women were 2.61 (95% CI: 2.59–2.64) and 1.92 (95% CI: 1.89–1.94) per 100,000, indicating a significant increase of 2.01% and 2.12%, respectively. Non-Hispanic Black men had the highest ASIR (4.25 per 100,000). Also, those aged 80–84 age group had the highest ASIR. During COVID-19, the ASIR of small intestine cancer decreased by 8.94% (5.06–12.81%).

**Conclusions:**

Small intestine cancer incidence raised in all sexes and ethnicities. Following COVID-19, reported cases declined, possibly due to pandemic-related diagnostic challenges. The impact of underdiagnosis on patient survival needs further investigations.

## Introduction

Despite being the largest segment of the digestive system, small intestine cancers are quite rare [1, 2]. Only 3% of all cancers of gastrointestinal system are due to the small intestine. Also, only 0.6% cancer numbers in the United States (US) arise from the small intestine [3, 4]. Small intestine adenocarcinoma and neuroendocrine tumors are the most common malignancies of the small intestine [5]. Due to its asymptomatic presentation and limited diagnostic tools, most cases are diagnosed at advanced stages, contributing to its poor prognosis [6]. The five-year survival rate of small intestine adenocarcinoma is about 35% [7].

In the past forty years, the incidence of small intestine cancer in the developed world has more than doubled [3]. The growing incidence of small intestine cancer is due to advancements in survival and diagnostic techniques, as well as lifestyle factors like rising obesity rates, smoking, and the increased consumption of alcohol and red/processed meats [3]. In 2023, more than 12 thousand new cases of small intestine cancer were estimated, with 2,070 cancer-related deaths in the US [8]. It is also influenced by sex, race/ethnicity, and age, with males having a higher likelihood of both diagnosis and mortality compared to females [3]. Also, Asians, Pacific Islanders, Native Americans, and Alaskans have the lowest risk of this malignancy [3].

The coronavirus disease 2019 (COVID-19) pandemic presented an unprecedented challenge to healthcare systems globally, leading to a significant reduction in most clinical activities. Colorectal cancer screening programs worldwide were paused [9], and the number of colonoscopies for other reasons were decreased [10, 11]. The pandemic led to a lower detection rate of cancer cases, with some countries experiencing up to a 70% drop in diagnoses [12]. So, it sounds that diagnosis of small intestine cancer has also been reduced. Nevertheless, it was not well-investigated in prior research.

A previous study assessed the changes in histology-specific incidence, treatment, and survival of small intestine cancer over 1973–2004 [13]. Other studies reported the epidemiology of some other types of small intestine cancers like gastrointestinal stromal tumors (GISTs) and carcinoid tumors in the US [14, 15]. However, they are only considered one specific subtype of small intestine cancers or are out-of-dated. Therefore, a more precise investigation of more recent incidence trends of small intestine cancer seems necessary. Our objective was to analyze the trends in small intestine cancer incidence and the impact of COVID-19 by age, sex, race/ethnicity, and histological subgroups from 2000 to 2020 in the US, utilizing data from the Surveillance, Epidemiology, and End Results (SEER) database.

## Methods

### Data source and ethics statement

The National Cancer Institute’s SEER program collects information on the occurrence and survival rates of cancer. SEER 22 encompasses approximately 48% of the population in the US. It provides data on patient survival rates and the stage of cancers when first diagnosed [16]. The registries consistently gather information on demographics, primary tumor location, morphology and stage, as well as data on initial treatments and follow-up [16]. For this study, we used the SEER 22 database to report the incidence rates and annual percent changes (APCs) of small intestine cancer over the study period [17].

We accessed the SEER 22 database under the SEER Research Data Agreement, covering data from 1975 to 2020 [18]. Cancer statistics were published in alignment with the SEER 22 guideline [19]. Given the nature of this study and the data which is available at http://seer.cancer.gov/data/, there was no requirement for institutional review board submission and do not require patient informed consent. Access to the SEER data was in accordance with the SEER data agreement. All data from SEER database were fully anonymized.

### Definitions

We used frequencies and percentages for reporting of cancer cases. Also, the incidence rates were provided as the number of cases per 100,000 population. The APCs of small intestine cancer over a specified time period exhibit variation at a consistent fraction of the previous year’s rate. The average annual percent changes (AAPCs) describe the average of various APCs over a defined time period. Non-Hispanic White (NHW), Non-Hispanic Black (NHB), and Hispanic were considered for race. Because of the limited number of cases, the race and ethnicity groups of American Indian/Alaska Native, Native Hawaiian, and Asian/Pacific Islander were only employed for calculating the parameters of all races and ethnicities. Patients with small intestine cancer were defined according to the International Classification of Diseases for Oncology version 3. The small intestine cancer morphologies were categorized into GIST (code 8936), neuroendocrine carcinoma (NEC) (codes 8013, 8041, 8240 to 8246, and 8249), and adenocarcinoma (codes 8140 to 8147, 8163, 8210, 8211, 8214, 8220, 8221, 8255, 8260 to 8263, 8310, 8480, 8481, 8490, 8510, 8570, and 8574 to 8576).

### Statistical analysis

Data from the SEER 22 Research Limited-Field Data database spanning 2000 to 2020 were used to calculate the age-standardised incidence rates (ASIRs) [17]. It was collected from the SEER*Stat software (version 8.4.1.2) [20]. ASIRs and 95% confidence intervals (CIs) were calculated by the Tiwari method and the 2000 US standard population [21] using the same software [20].

The estimatation of the APCs, AAPCs [22], joinpoint regression modeling, parallelism test, and coincident test [23] for ASIR [24], was conducted using the Joinpoint Regression Program (version 5.0.2) [25]. Due to the potential for bias in cancer incidence estimates from the 2020 data because of the COVID-19 pandemic, this year’s data was omitted from Joinpoint trends and only presented in graphs. To calculate the ASIR of small intestine cancer APCs, the best fit of least-squares regression lines on the natural logarithm of the ASIR was used, with the diagnosis year as a regressor variable. Both the minimum number of observations between two joinpoints and the minimum number of observations from the joinpoint to either end of the data were set to two. In joinpoint regression, the dependent and independent variables were the year of diagnosis and ASIRs, respectively. Other parameters like sex and race/ethnicity were considered as by-variables [26]. For selecting models, the weighted Bayesian Information Criteria technique was employed [27]. To obtain the 95% CIs of AAPCs, the empirical quantile method was used [28]. The parallelism test was used in a pairwise comparison to assess whether the trends of the two groups were similar over time [23]. Moreover, a pairwise comparison was carried out using the coincidence test to assess whether the rates of the two groups were identical throughout the study period.

### Ethics statement

Given the nature of this study and the data which is available at http://seer.cancer.gov/data/, there was no requirement for institutional review board submission and do not require patient informed consent. Access to the SEER data was in accordance with the SEER data agreement. All data from SEER database were fully anonymized.

## Results

### Small intestine cancer

#### Overall incidence

**Fig 1:**
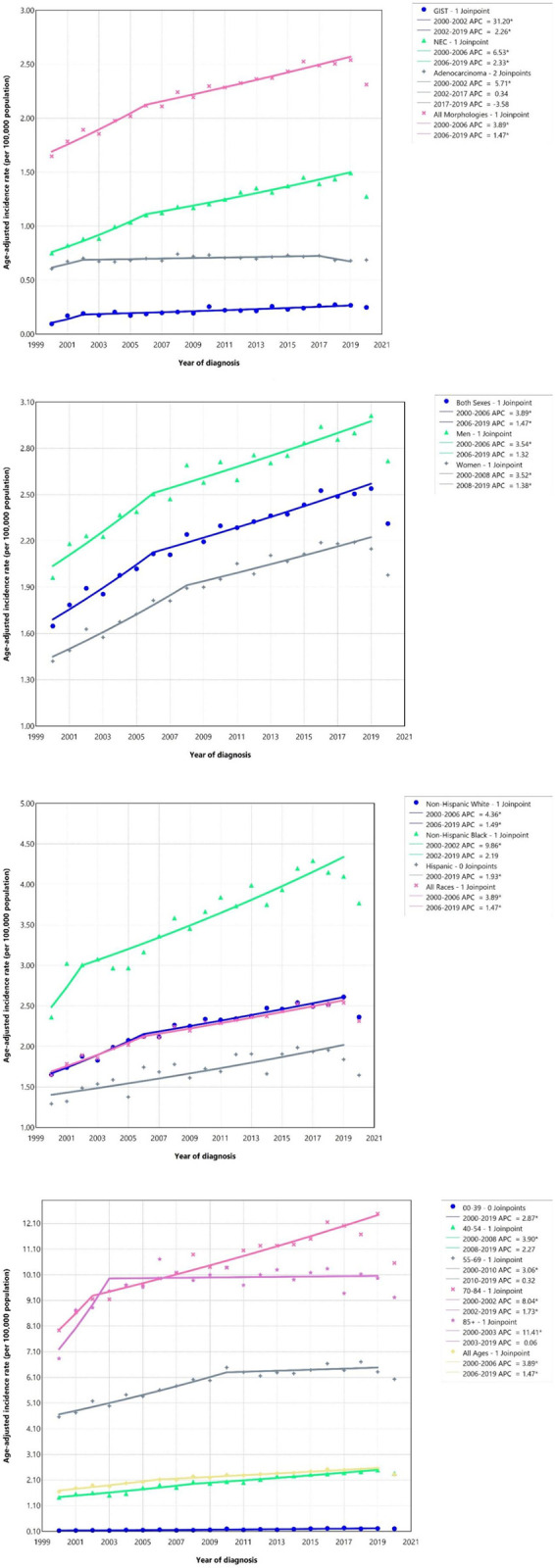
Age-adjusted incidence rate of small intestine cancer over 2000–2019 and in 2020 in the United States, by histologic type, sex, race/ethnicity, and age. APC annual percent change. NEC: Neuroendocrine carcinoma; GIST: Gastrointestinal stromal tumor. *Represent p-value less than 0.05.

From 2000 to 2019, a total of 67,815 cases of small intestine cancer were reported in the US among all ages. The most common subtype was NEC (54.26%). The majority of cases were among those aged 55 to 69 years (38.08%), men (53.10%), and NHWs (69.07%), living in metropolitan counties (87.83%), and with median income over $65,000 per year (60.38%) (Table 1). From 2000 to 2019, the ASIR of small intestine cancer per 100,000 population for men and women were 2.61 (95% CI: 2.59, 2.64) and 1.92 (1.89, 1.94), respectively, indicating 2.01% (1.43, 2.70) and 2.12% (1.82, 2.46) increases among men and women, respectively. The highest ASIR of small intestine cancer per 100,000 was among NHB men (4.25 [4.13, 4.38]) and the highest AAPC was among NHB women (3.39% [2.04, 4.80]) (Table 2 and Fig 1).

**Table 1:** Demographic characteristics of cases with small intestine cancer by the year of diagnosis.

Characteristics	2000–2004 N(%)	2005–2009 N(%)	2010–2014 N(%)	2015–2019 N(%)	2019–2020 N(%)	2000–2019 N(%)	2000–2020 N(%)
***Sex***							
Male	6311 (52.74)	8127 (53.04)	9812 (52.79)	11758 (53.59)	4851 (54.68)	36008 (53.10)	38343 (53.20)
Female	5656 (47.26)	7196 (46.96)	8774 (47.21)	10181 (46.41)	4021 (45.32)	31807 (46.90)	33737 (46.80)
***Age***							
≤39	536 (4.48)	577 (3.77)	675 (3.63)	876 (3.99)	344 (3.88)	2664 (3.93)	2831 (3.93)
40–54	2290 (19.14)	3056 (19.94)	3490 (18.78)	3743 (17.06)	1490 (16.79)	12579 (18.55)	13300 (18.45)
55–69	4065 (33.97)	5630 (36.74)	7427 (39.96)	8696 (39.64)	3413 (38.47)	25818 (38.07)	27493 (38.14)
70–84	4220 (35.26)	4923 (32.13)	5672 (30.52)	7173 (32.70)	3053 (34.41)	21988 (32.42)	23414 (32.48)
≥85	856 (7.15)	1137 (7.42)	1322 (7.11)	1451 (6.61)	572 (6.45)	4766 (7.03)	5042 (7.00)
***Median Income per year*** ^***a***^							
< $35,000	46 (0.38)	87 (0.57)	133 (0.72)	112 (0.51)	39 (0.44)	378 (0.56)	400 (0.55)
$35,000 - $49,999	793 (6.63)	1311 (8.56)	2182 (11.74)	1853 (8.45)	662 (7.46)	6139 (9.05)	6470 (8.98)
$50,000 - $64,999	3013 (25.18)	4435 (28.94)	7090 (38.15)	5810 (26.48)	1921 (21.65)	20348 (30.01)	21263 (29.50)
≥ $65,000	8113 (67.79)	9488 (61.92)	9181 (49.40)	14164 (64.56)	6250 (70.45)	40946 (60.38)	43943 (60.96)
Unknown ^b^	2 (0.02)	2 (0.01)	0 (0.00)	0 (0.00)	0 (0.00)	4 (0.01)	4 (0.01)
***Urban/Rural***							
Metropolitan counties	10374 (86.69)	13423 (87.60)	16333 (87.88)	19433 (88.58)	7864 (88.64)	59563 (87.83)	63344 (87.88)
Non-Metropolitan counties	1584 (13.24)	1892 (12.35)	2250 (12.11)	2501 (11.40)	1004 (11.32)	8227 (12.13)	8709 (12.08)
Other ^c^	9 (0.07)	8 (0.05)	3 (0.02)	5 (0.02)	4 (0.05)	25 (0.04)	27 (0.04)
***Race/ Ethnicities***							
Hispanic	999 (8.69)	1448 (9.86)	1979 (11.16)	2660 (12.83)	1062 (12.78)	7086 (10.96)	7603 (11.08)
NHB	1742 (15.16)	2256 (15.36)	2994 (16.88)	3734 (18.01)	1506 (18.13)	10726 (16.59)	11462 (16.70)
NHW	8750 (76.15)	10986 (74.79)	12766 (71.97)	14340 (69.16)	5739 (69.09)	46842 (72.45)	49568 (72.22)
***Type of Reporting Source***							
Autopsy only	57 (0.48)	62 (0.40)	46 (0.25)	34 (0.15)	6 (0.07)	199 (0.29)	202 (0.28)
Death certificate only	51 (0.43)	47 (0.31)	61 (0.33)	108 (0.49)	48 (0.54)	267 (0.39)	288 (0.40)
Hospital inpatient/outpatient or clinic	11510 (96.18)	14446 (94.28)	17261 (92.87)	19955 (90.96)	8018 (90.37)	63172 (93.15)	67040 (93.01)
Laboratory only (hospital or private)	70 (0.58)	138 (0.90)	251 (1.35)	372 (1.70)	167 (1.88)	831 (1.23)	914 (1.27)
Nursing/convalescent home/hospice	7 (0.06)	5 (0.03)	14 (0.08)	11 (0.05)	3 (0.03)	37 (0.05)	37 (0.05)
Other hospital outpatient unit or surgery center (2006+)	191 (1.60)	351 (2.29)	662 (3.56)	939 (4.28)	423 (4.77)	2143 (3.16)	2345 (3.25)
Physician’s office/private medical practitioner	71 (0.59)	178 (1.16)	139 (0.75)	205 (0.93)	81 (0.91)	593 (0.87)	634 (0.88)
Radiation treatment or medical oncology center (2006+)	10 (0.08)	96 (0.63)	152 (0.82)	315 (1.44)	126 (1.42)	573 (0.84)	620 (0.86)

**Abbreviations**: **NHW**: Non-Hispanic White; **NHB**: Non-Hispanic Black

^a^ Median Household Income Adjusted to 2021 Inflation

^b^ Missing/No Match/Not 1990–2021

^c^ Missing/No Match/Not 1990-2021/Alaska or Hawaii—Entire State

**Table 2:** Counts and age-standardized rate of small intestine cancer incidence per 100,000 and average annual percent change from 2000 to 2019 in the United States, by age, sex, and race.

**All race/ethnicities**						
**Age group (years)**	**Men**			**Women**		
	**Case (%)**	**ASIR (95% CI)**	**AAPC (95% CI)**	**Case (%)**	**ASIR (95% CI)**	**AAPC (95% CI)**
**All**	36008 (53.10)	2.61 (2.59, 2.64)	2.01 (1.43, 2.7)	31807(46.90)	1.92 (1.89, 1.94)	2.12 (1.82, 2.46)
**0 to 39**	1352 (1.99)	0.17 (0.16, 0.18)	2.22 (1.19, 3.37)	1312 (1.93)	0.17 (0.16, 0.18)	3.63 (2.75, 4.68)
**40 to 54**	6719 (9.91)	2.16 (2.11, 2.21)	2.54 (1.87, 3.29)	5860 (8.64)	1.83 (1.79, 1.88)	2.98 (1.96, 4.48)
**55 to 69**	14245 (21.01)	6.94 (6.83, 7.06)	1.5 (0.83, 2.45)	11573(17.07)	5.12 (5.03, 5.21)	2.04 (1.44, 2.85)
**70 to 84**	11624 (17.14)	13.09 (12.86, 13.34)	1.92 (1.55, 2.33)	10364(15.28)	8.78 (8.62, 8.96)	1.96 (1.51, 2.47)
**+85**	2068 (3.05)	12.96 (12.4, 13.53)	0.43 (-0.43, 1.7)	2698 (3.98)	8.24 (7.93, 8.55)	2.41 (1.62, 3.74)
**Hispanic**						
**Age groups**	**Men**			**Women**		
	**Case (%)**	**ASIR (95% CI)**	**AAPC (95% CI)**	**Case (%)**	**ASIR (95% CI)**	**AAPC (95% CI)**
**All**	3688 (52.05)	1.99 (1.92, 2.06)	2 (1.28, 2.97)	3398 (47.95)	1.54 (1.49, 1.6)	1.82 (0.98, 3.07)
**0 to 39**	267 (3.77)	0.13 (0.11, 0.14)	2.59 (0.35, 5.36)	263 (3.71)	0.13 (0.12, 0.15)	2.95 (0.48, 6.22)
**40 to 54**	926 (13.07)	1.56 (1.46, 1.66)	3.11 (1.82, 4.84)	771 (10.88)	1.3 (1.21, 1.39)	2.56 (1.17, 4.34)
**55 to 69**	1427 (20.14)	5.27 (5, 5.56)	1.5 (0.23, 3.25)	1161 (16.38)	3.79 (3.57, 4.01)	2.12 (0.88, 3.75)
**70 to 84**	915 (12.91)	9.99 (9.34, 10.66)	2.28 (0.76, 4.29)	998 (14.08)	7.86 (7.38, 8.36)	0.52 (-0.95, 3.37)
**+85**	153 (2.16)	11.11 (9.42, 13.02)	-0.41 (-2.9, 3.07)	205 (2.89)	8.01 (6.95, 9.19)	-2.1 (-4.21, 0.46)
**NHB**						
**Age groups**	**Men**			**Women**		
	**Case (%)**	**ASIR (95% CI)**	**AAPC (95% CI)**	**Case (%)**	**ASIR (95% CI)**	**AAPC (95% CI)**
**All**	5316 (49.56)	4.25 (4.13, 4.38)	1.97 (1.22, 2.9)	5410 (50.44)	3.16 (3.08, 3.25)	3.39 (2.04, 4.8)
**0 to 39**	225 (2.10)	0.25 (0.22, 0.29)	1.79 (-1.1, 5.1)	260 (2.42)	0.27 (0.23, 0.3)	5.13 (3.12, 7.77)
**40 to 54**	1216 (11.34)	3.57 (3.37, 3.78)	2.16 (0.46, 4.11)	1219 (11.36)	3.16 (2.98, 3.34)	2.32 (0.62, 3.71)
**55 to 69**	2279 (21.25)	11.73 (11.25, 12.23)	1.71 (0.7, 2.98)	2185 (20.37)	8.98 (8.6, 9.36)	2.62 (1.18, 4.96)
**70 to 84**	1404 (13.09)	20.84 (19.75, 21.97)	2.3 (1.09, 3.85)	1454 (13.55)	13.7 (13, 14.42)	3.02 (1.61, 4.92)
**+85**	192 (1.79)	20.11 (17.37, 23.17)	0.56 (-2.59, 4.86)	292 (2.72)	12.08 (10.74, 13.55)	3.07 (-4.35, 10.95)
**NHW**						
**Age groups**	**Men**			**Women**		
	**Case (%)**	**ASIR (95% CI)**	**AAPC (95% CI)**	**Case (%)**	**ASIR (95% CI)**	**AAPC (95% CI)**
**All**	25223 (53.85)	2.63 (2.6, 2.66)	2.2 (1.81, 2.8)	21619(46.15)	1.91 (1.88, 1.94)	2.21 (1.91, 2.52)
**0 to 39**	753 (1.61)	0.19 (0.17, 0.2)	2.56 (1.1, 4.14)	697 (1.49)	0.17 (0.16, 0.19)	2.79 (1.69, 3.98)
**40 to 54**	4202 (8.97)	2.2 (2.13, 2.27)	3.2 (2.31, 4.2)	3597 (7.68)	1.87 (1.81, 1.93)	3.1 (2.03, 4.28)
**55 to 69**	9853 (21.03)	6.9 (6.76, 7.03)	1.67 (0.7, 2.87)	7720(16.48)	5.06 (4.95, 5.18)	2.12 (1.65, 2.72)
**70 to 84**	8778 (18.74)	13.17 (12.9, 13.45)	1.99 (1.52, 2.5)	7493(16.00)	8.67 (8.47, 8.86)	1.81 (1.37, 2.29)
**+85**	1637 (3.49)	13.02 (12.4, 13.67)	0.57 (-0.49, 1.97)	2112 (4.51)	8.13 (7.78, 8.48)	2.88 (1.37, 4.69)

Abbreviations: NHW: Non-Hispanic White; NHB: Non-Hispanic Black; ASIR: Age-standardized incidence rate; CI: Confidence interval, AAPC: Average annual percent change.

Over 2015–2019, 21,939 cases of small intestine cancer were reported in the US. The majority of them were among those aged 55 to 69 years (39.63%), men (53.59%), and NHWs (65.36%). The ASIRs per 100,000 population were 2.91 (2.86, 2.97) for men and 2.17 (2.12, 2.21) for women which had no significant AAPCs. By race/ethnicity, NHB men had the highest ASIR (4.75 [4.52, 4.99]) (S1 Table). The results of parallel and identical trends of small intestine cancer over 2000–2019 are provided in S2 and S3 Tables, respectively.

#### Men

Over 2000–2019, a total of 36,008 cases of small intestine cancer were diagnosed in men. NEC was the most common subtype (53.44%). Moreover, most reported cases aged from 55 to 69 years (39.56%) and were NHWs (70.04%). Cases aged 70 to 84 years had the highest ASIR among all age groups (13.09 [12.86, 13.34]). Additionally those between 40 and 54 years exhibited the greatest increase in ASIRs compared to other age groups (AAPC: 2.54%; 1.87, 3.29) (Table 2).

Of all the reported cases, 10.24% were among Hispanic men, the majority of cases were between 55 and 69 years (38.69%). The overall ASIR per 100,000 population was 1.99 (1.92, 2.06) with cases aged above 85 years having the highest ASIR (11.11 [9.42, 13.02]). All age groups showed significant increases in ASIRs from 2000 to 2019 except for those aged above 85 years. Furthermore, individuals between 40 and 54 years had the greatest increase among all other age groups (3.11% [1.82, 4.84]) (Table 2).

Of all men, 14.76% were NHBs. The majority of cases (42.87%) were between 55 and 69 years. The ASIR per 100,000 population for NHB men was 4.25 (4.13, 4.38), and those aged 70 to 84 years had the highest ASIR (20.84 [19.75, 21.97]). Those aged 70 to 84 years had the greatest increase in ASIRs (AAPC: 2.30%; [1.09, 3.85]) (Table 2).

NHWs consisted of 70.05% of the total cases. The majority of NHW men were between 55 and 69 years (39.06%). The overall ASIR per 100,000 population was 2.63 (2.60, 2.66) with cases aged 70 to 84 years having the highest ASIR (13.17 [12.90, 13.45]). All age groups experienced a significant increase in ASIRs over 2000–2019 except for those above 85 years (Table 2).

#### Women

A total of 31,807 cases of small intestine cancer were reported over 2000–2019 among women in the US. Most of the cases were NEC (55.19%). The majority of cases occurred in NHWs (67.97%) and those aged 55–69 years (36.39%). Cases aged 70 to 84 years had the highest ASIR among all age groups (8.78 [8.62, 8.96]). All age groups showed an increase in ASIRs over 2000–2019 with cases below 39 years having the greatest increase (AAPC: 3.63%; 2.75, 4.68) (Table 2).

Of all the women, 10.68% were Hispanic and the majority of cases were between 55 and 69 years (34.17%). The ASIR per 100,000 population in Hispanic women was 1.54 (1.49, 1.60) with individuals over 85 years having the highest ASIR (8.01 [6.95, 9.19]). Those aged <39 years had the greatest increase in ASIRs among all age groups over 2000–2019 (AAPC: 2.95%; 0.48, 6.22) (Table 2).

Among women, 17.00% of reported cases were NHBs and the majority of cases (40.38%) were between 55 and 69 years. The ASIR per 100,000 population in NHB women was 3.16 (3.08, 3.25). Cases aged between 70 and 84 years had the highest ASIR (13.70 [13.00, 14.42]). All age groups showed significant increases over 2000–2019 except for those above 85 years, with those aged <39 years having the highest AAPC (5.13% [3.12, 7.77]) (Table 2).

The majority of reported females were NHWs (67.96%) and aged 55–69 years (35.70%). The ASIR per 100,000 population in NHW women was 1.91 (1.88, 1.94). Those aged 70 to 84 years had the highest ASIR (8.67 [8.47, 8.86]). Over 2000–2019, overall ASIR showed a significant increase in NHW women among all age groups with those aged 40 to 54 years having the greatest AAPC (3.10% [2.03, 4.28]) (Table 2).

#### Age and sex patterns

**Fig 2:**
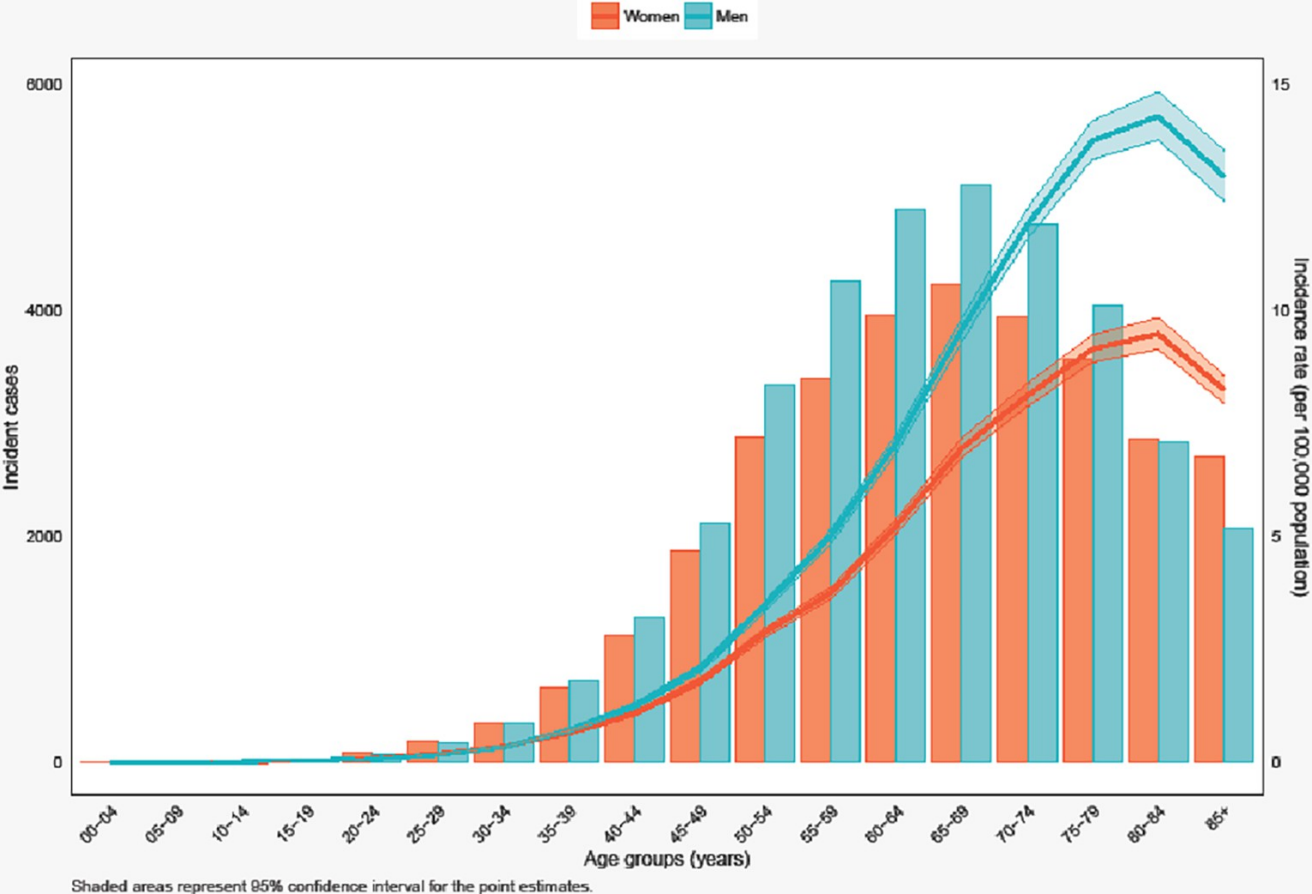
Incident numbers and incidence rate (per 100,000 population) of small intestine cancer in the United States among males and females in each age group.

The incident cases of small intestine cancer increased with advancing age among both males and females and peaked at 65–69 age group. Similarly, the incidence rates increased with age and peaked at 80–84 age group. Generally, males had higher incidence rates of small intestine cancer than females (Fig 2).

### COVID-19 impacts

There was a significant decrease in the ASIRs of small intestine cancer across all races/ethnicities in both sexes within all age groups (PC: -8.94% [-12.81, -5.06]) and for males (PC: -9.75% [-14.95, -4.55]) and females (PC: -7.89% [-13.73, -2.04]) from 2019 to 2020 (Table 3).

**Table 3:** Percent change in all age-adjusted incidence rates of small intestine cancer from 2019 to 2020, by race and sex in the United States, using the November 2022 data submission.

Races/ethnicities	Sex	2019 ASIR (95% CI)	2020 ASIR (95% CI)	PC (95% CI)
All	Both	2.54 (2.47, 2.62)	2.31 (2.24, 2.38)	-8.94 (-12.81, -5.06)
All	Male	3.01 (2.89, 3.14)	2.72 (2.61, 2.83)	-9.75 (-14.95, -4.55)
All	Female	2.15 (2.06, 2.24)	1.98 (1.89, 2.07)	-7.89 (-13.73, -2.04)
Hispanic	Both	1.84 (1.68, 2.01)	1.64 (1.5, 1.8)	-10.56 (-21.72, 0.6)
Hispanic	Male	2.14 (1.89, 2.42)	1.81 (1.58, 2.07)	-15.36 (-30.54, -0.17)
Hispanic	Female	1.6 (1.4, 1.81)	1.54 (1.36, 1.74)	-3.71 (-20.67, 13.25)
NHB	Both	4.1 (3.81, 4.41)	3.77 (3.5, 4.06)	-8.04 (-17.59, 1.51)
NHB	Male	4.86 (4.37, 5.39)	4.34 (3.89, 4.83)	-10.7 (-24.03, 2.62)
NHB	Female	3.56 (3.21, 3.95)	3.36 (3.02, 3.74)	-5.65 (-19.49, 8.19)
NHW	Both	2.61 (2.52, 2.71)	2.36 (2.27, 2.46)	-9.52 (-14.42, -4.62)
NHW	Male	3.13 (2.98, 3.29)	2.84 (2.7, 2.99)	-9.3 (-15.78, -2.82)
NHW	Female	2.16 (2.04, 2.29)	1.95 (1.83, 2.07)	-9.77 (-17.28, -2.25)

Abbreviations: NHW: Non-Hispanic White; NHB: Non-Hispanic Black; ASIR: Age-standardized incidence rate; CI: Confidence interval, PC: percent change.

### Adenocarcinoma

#### Overall incidence

From 2000 to 2019, there were 21,086 adenocarcinoma cases in all age groups in the US. The majority of cases were men (54.14%), NHWs (66.72%), and between 70 and 84 years (37.91%). The ASIR per 100,000 population was 0.85 (0.84, 0.87) for men and 0.58 (0.57, 0.59) for women. The AAPCs for men and women were 0.49% (0.16, 0.85) and 0.02% (-0.64, 0.70), respectively. NHB men had the highest ASIR per 100,000 population (1.53 [1.46, 1.61]) and Hispanic men had the greatest AAPC (1.20% [0.13, 2.56]) (Table 4, S1–S3 Figs).

**Table 4:** Counts and age-standardized rate of adenocarcinoma incidence per 100,000 and average annual percent change from 2000 to 2019 in the United States, by age, sex, and race.

**All race/ethnicities**							
**Age group (years)**	**Men**			**Women**			
	**Case (%)**	**ASIR (95% CI)**	**AAPC (95% CI)**	**Case (%)**	**ASIR (95% CI)**		**AAPC (95% CI)**
**All**	11416 (54.14)	0.85 (0.84, 0.87)	0.49 (0.16, 0.85)	9670 (45.86)	0.58 (0.57, 0.59)		0.02 (-0.64, 0.7)
**0 to 39**	378 (1.79)	0.05 (0.04, 0.05)	-0.96 (-2.67, 0.74)	309 (1.47)	0.04 (0.04, 0.04)		1.75 (0.28, 3.39)
**40 to 54**	1810 (8.58)	0.58 (0.56, 0.61)	0.88 (-0.12, 1.93)	1271 (6.03)	0.4 (0.38, 0.42)		-0.01 (-1.05, 1.05)
**55 to 69**	4045 (19.18)	1.98 (1.91, 2.04)	0.07 (-0.62, 0.88)	3094 (14.67)	1.37 (1.32, 1.42)		-0.89 (-1.77, 0.33)
**70 to 84**	4238 (20.10)	4.79 (4.65, 4.94)	0.66 (-0.13, 1.52)	3755 (17.81)	3.17 (3.07, 3.27)		-0.1 (-0.82, 0.61)
**+85**	945 (4.48)	5.92 (5.55, 6.31)	0.74 (-0.21, 1.87)	1241 (5.89)	3.79 (3.58, 4.01)		2.75 (1.54, 4.72)
**Hispanic**							
**Age groups**	**Men**			**Women**			
	**Case (%)**	**ASIR (95% CI)**	**AAPC (95% CI)**	**Case (%)**	**ASIR (95% CI)**	**AAPC (95% CI)**	
**All**	1195 (53.52)	0.70 (0.66, 0.74)	1.2 (0.13, 2.56)	1038 (46.48)	0.5 (0.47, 0.54)	-2.1 (-3.93, 0.01)	
**0 to 39**	72 (3.22)	0.03 (0.03, 0.04)	-1.28 (-4.06, 1.59)	65 (2.91)	0.03 (0.03, 0.04)	2.21 (-2.71, 8.36)	
**40 to 54**	263 (11.78)	0.44 (0.39, 0.5)	0.97 (-2.09, 4.91)	156 (6.99)	0.26 (0.22, 0.31)	-1.16 (-4.78, 2.9)	
**55 to 69**	421 (18.85)	1.58 (1.43, 1.74)	0.18 (-1.73, 2.73)	330 (14.78)	1.08 (0.97, 1.21)	-1.82 (-5.21, 2.35)	
**70 to 84**	368 (16.48)	4.07 (3.66, 4.51)	1.71 (-0.27, 4.42)	384 (17.20)	3.04 (2.74, 3.36)	-3.4 (-6.68, 0.86)	
**+85**	71 (3.18)	5.16 (4.03, 6.51)	N/A	103 (4.61)	4.03 (3.29, 4.88)	-3.67 (-6.64, -0.31)	
**NHB**							
**Age groups**	**Men**			**Women**			
	**Case (%)**	**ASIR (95% CI)**	**AAPC (95% CI)**	**Case (%)**	**ASIR (95% CI)**	**AAPC (95% CI)**	
**All**	1850 (50.37)	1.53 (1.46, 1.61)	0.56 (-0.63, 1.93)	1823 (49.63)	1.09 (1.04, 1.14)	0.6 (-0.54, 1.91)	
**0 to 39**	84 (2.29)	0.1 (0.08, 0.12)	N/A	87 (2.37)	0.09 (0.07, 0.11)	3.15 (0.27, 6.67)	
**40 to 54**	384 (10.45)	1.13 (1.02, 1.25)	-0.02 (-2.28, 2.37)	366 (9.96)	0.95 (0.85, 1.05)	-1.62 (-4.84, 0.51)	
**55 to 69**	766 (20.85)	3.95 (3.68, 4.25)	0.16 (-1.71, 2.54)	667 (18.16)	2.73 (2.53, 2.95)	0.25 (-1.35, 2.14)	
**70 to 84**	520 (14.16)	7.77 (7.11, 8.48)	1.12 (-0.84, 3.54)	557 (15.16)	5.29 (4.85, 5.74)	0.74 (-0.86, 2.58)	
**+85**	96 (2.61)	10.06(8.14, 12.28)	N/A	146 (3.97)	6.04 (5.1, 7.11)	-1.66 (-5.91, 3.04)	
**NHW**							
**Age groups**	**Men**			**Women**			
	**Case (%)**	**ASIR (95% CI)**	**AAPC (95% CI)**	**Case (%)**	**ASIR (95% CI)**	**AAPC (95% CI)**	
**All**	7727 (54.93)	0.82 (0.8, 0.84)	0.47 (-0.16, 1.19)	6341 (45.07)	0.54 (0.53, 0.55)	0.24 (-0.43, 0.93)	
**0 to 39**	192 (1.36)	0.05 (0.04, 0.05)	0.17 (-2.52, 2.89)	136 (0.97)	0.03 (0.03, 0.04)	0.16 (-2.83, 3.14)	
**40 to 54**	1038 (7.38)	0.55 (0.51, 0.58)	1.01 (-0.4, 2.41)	670 (4.76)	0.35 (0.32, 0.37)	-0.22 (-2.23, 1.57)	
**55 to 69**	2650 (18.84)	1.86 (1.79, 1.93)	0.09 (-1.01, 1.33)	1949 (13.85)	1.28 (1.22, 1.34)	-0.29 (-1.24, 0.76)	
**70 to 84**	3113 (22.13)	4.68 (4.52, 4.85)	0.4 (-0.63, 1.49)	2641 (18.77)	3.04 (2.92, 3.16)	0.12 (-0.72, 1)	
**+85**	734 (5.22)	5.84 (5.42, 6.28)	0.74 (-0.72, 2.46)	945 (6.72)	3.64 (3.41, 3.88)	3.3 (1.68, 5.58)	

Abbreviations: NHW: Non-Hispanic White; NHB: Non-Hispanic Black; ASIR: Age-standardized incidence rate; CI: Confidence interval, AAPC: Average annual percent change; N/A: Not available.

#### Men

A total of 11,416 cases of adenocarcinoma were reported in men over 2000–2019. The majority of cases were NHWs (67.69%) and between 70 and 84 years (37.12%). The greatest overall ASIR per 100,000 population among men belonged to those aged above 85 years (5.92 [5.55, 6.31]) (Table 4).

Of all the reported cases 1,195 (10.47%) were Hispanics. The majority of them were between 55 and 69 years (35.23%). The overall ASIR per 100,000 population was 0.70 (0.66, 0.74). Cases over 85 years had the highest ASIR among other age groups 5.16 (4.03, 6.51) (Table 4).

NHBs consisted of 16.21% of the men with adenocarcinoma. The majority of NHBs were between 55 and 69 years (41.41%). The overall ASIR per 100,000 population was 1.53 (1.46, 1.61), with those above 85 years having the highest ASIR (10.06 [8.14, 12.28]) (Table 4).

There were 7,727 (67.69%) cases of NHWs among men with adenocarcinoma. The majority of the cases were between 70 and 84 years (40.29%). The overall ASIR per 100,000 population was 0.82 (0.80, 0.84) with cases over 85 years having the highest ASIR (5.84 [5.42, 6.28]). The AAPCs were not significant in none of the age groups (Table 4).

#### Women

Among women, a total of 9,670 adenocarcinoma cases were reported. The majority of these cases were among NHWs (65.57%) and those aged 70–84 years (38.83%). The greatest overall ASIR per 100,000 population was among those aged >85 years (3.79 [3.58, 4.01]). Notably, only those <39 and >85 years had significant increases in ASIRs over 2000–2019 (Table 4).

Among all reported cases, 1,038 (10.73%) were among Hispanic individuals. The majority of cases were between 70 and 84 years (36.99%). The overall ASIR per 100,000 population for this group was 0.50 (0.47, 0.54), with those >85 years having the highest ASIR (4.03 [3.29, 4.88]). The greatest significant decline in ASIR from 2000–2019 was observed among those >85 years (AAPC: -3.67%; -6.64, -0.31) (Table 4).

NHBs constituted 18.85% of the cases of adenocarcinoma in women. A significant portion of NHB cases fell within the 55 to 69-year age group (36.59%). The overall ASIR per 100,000 population for NHB women was 1.09 (1.04, 1.14), with cases >85 years having the highest ASIR (6.04 [5.10, 7.11]). Furthermore, the greatest increase in ASIR was among those <39 years (AAPC: 3.15%; 0.27, 6.67) (Table 4).

Among women with adenocarcinoma, there were 6,341 (65.57%) reported cases of NHWs. The majority of NHW cases were in the 70–84 age group (41.65%). The overall ASIR per 100,000 population for this group was 0.54 (0.53, 0.55), with cases >85 years having the highest ASIR (3.64 [3.41, 3.88]). Over the 2000–2019 period, those >85 years had the greatest increase in ASIRs (AAPC: 3.30%; 1.68, 5.58) (Table 4).

#### Age and sex patterns

Between 2000 and 2019, across all races and ethnicities, the incidence rate of adenocarcinoma showed minimal variation in both males and females up to the 25–29 age groups. Then, both groups experienced an exponential increase. The incidence rates peaked at 80–84 and >85 age groups in females and males, respectively. The highest incident cases were in the 70–74 age group for men and 75–79 age group for women (S4 Fig).

### NEC

#### Overall incidence

Over 2000–2019, there were a total of 36,801 NEC cases. The majority of cases were in men (52.29%), NHWs (70.74%), and individuals aged 55 to 69 years (41.18%). The ASIR per 100,000 population was 1.37 (1.35, 1.39) for men and 1.06 (1.05, 1.08) for women. The AAPCs for men and women were 3.35% (2.92, 4.03) and 3.72% (3.31, 4.33), respectively. NHB men had the highest ASIR (2.27 [2.19, 2.36]) and NHB women had the greatest increase in ASIRs (AAPC: 4.27%; 3.24, 5.61) (Table 5, S5–S7 Figs).

**Table 5:** Counts and age-standardized rate of neuroendocrine carcinoma incidence per 100,000 and average annual percent change from 2000 to 2019 in the United States, by age, sex, and race.

**All race/ethnicities**						
**Age group (years)**	**Men**			**Women**		
	**Case (%)**	**ASIR (95% CI)**	**AAPC (95% CI)**	**Case (%)**	**ASIR (95% CI)**	**AAPC (95% CI)**
**All**	19246 (52.29)	1.37 (1.35, 1.39)	3.35 (2.92, 4.03)	17555 (47.71)	1.06 (1.05, 1.08)	3.72 (3.31, 4.33)
**0 to 39**	622 (1.69)	0.08 (0.07, 0.09)	4.45 (2.46, 6.88)	685 (1.86)	0.09 (0.08, 0.09)	4.88 (3.27, 6.93)
**40 to 54**	3802 (10.34)	1.22 (1.18, 1.26)	3.47 (2.61, 4.48)	3703 (10.06)	1.16 (1.12, 1.19)	4.28 (2.69, 6.63)
**55 to 69**	8240 (22.39)	4.01 (3.93, 4.1)	2.66 (2.12, 3.48)	6918 (18.79)	3.06 (2.99, 3.13)	3.27 (2.64, 4.08)
**70 to 84**	5830 (15.85)	6.55 (6.38, 6.72)	3.89 (2.66, 5.22)	5275 (14.33)	4.48 (4.36, 4.61)	3.8 (3.02, 4.95)
**+85**	752 (2.05)	4.71 (4.38, 5.06)	0.99 (-0.6, 3.01)	974 (2.64)	2.97 (2.79, 3.17)	0.84 (-0.53, 2.43)
**Hispanic**						
**Age groups**	**Men**			**Women**		
	**Case (%)**	**ASIR (95% CI)**	**AAPC (95% CI)**	**Case (%)**	**ASIR (95% CI)**	**AAPC (95% CI)**
**All**	1802 (50.64)	0.94 (0.89, 0.99)	2.89 (1.43, 4.94)	1756 (49.36)	0.78 (0.74, 0.82)	3.86 (2.67, 5.55)
**0 to 39**	106 (2.98)	0.05 (0.04, 0.06)	3.69 (0.05, 8.91)	113 (3.18)	0.06 (0.05, 0.07)	N/A
**40 to 54**	478 (13.44)	0.81 (0.73, 0.88)	4.08 (2.07, 6.96)	464 (13.04)	0.78 (0.71, 0.86)	2.96 (1.55, 4.75)
**55 to 69**	751 (21.11)	2.76 (2.56, 2.97)	2.35 (0.6, 4.91)	645 (18.12)	2.11 (1.95, 2.28)	4.6 (3.36, 6.45)
**70 to 84**	419 (11.78)	4.51 (4.09, 4.97)	2.91 (0.39, 6.47)	469 (13.18)	3.67 (3.35, 4.02)	5.79 (3.04, 9.75)
**+85**	48 (1.35)	3.49 (2.57, 4.62)	N/A	65 (1.82)	2.54 (1.96, 3.24)	-0.49 (-3.81, 4.36)
**NHB**						
**Age groups**	**Men**			**Women**		
	**Case (%)**	**ASIR (95% CI)**	**AAPC (95% CI)**	**Case (%)**	**ASIR (95% CI)**	**AAPC (95% CI)**
**All**	2924 (48.48)	2.27 (2.19, 2.36)	3.37 (2.29, 5.15)	3107 (51.52)	1.8 (1.73, 1.86)	4.27 (3.24, 5.61)
**0 to 39**	104 (1.73)	0.11 (0.09, 0.14)	4.34 (1.36, 8.3)	155 (2.57)	0.16 (0.13, 0.19)	6.9 (3.57, 11.74)
**40 to 54**	699 (11.59)	2.05 (1.9, 2.21)	3.41 (1.62, 5.57)	753 (12.48)	1.95 (1.81, 2.09)	5.81 (4.36, 7.75)
**55 to 69**	1317 (21.84)	6.77 (6.41, 7.15)	2.77 (1.36, 4.62)	1323 (21.93)	5.45 (5.16, 5.75)	3.47 (2.63, 4.54)
**70 to 84**	739 (12.26)	10.87 (10.09, 11.69)	3.46 (2.03, 5.36)	773 (12.81)	7.25 (6.74, 7.78)	4.96 (1.36, 10.69)
**+85**	65 (1.08)	6.81 (5.25, 8.68)	-0.43 (-4.99, 5.71)	103 (1.71)	4.26 (3.48, 5.17)	0.86 (-2.92, 5.92)
**NHW**						
**Age groups**	**Men**			**Women**		
	**Case (%)**	**ASIR (95% CI)**	**AAPC (95% CI)**	**Case (%)**	**ASIR (95% CI)**	**AAPC (95% CI)**
**All**	13855 (53.22)	1.43 (1.4, 1.45)	3.7 (3.16, 4.51)	12177 (46.78)	1.09 (1.07, 1.11)	3.38 (2.7, 4.06)
**0 to 39**	377 (1.45)	0.09 (0.08, 0.1)	4.59 (2.72, 6.88)	385 (1.47)	0.1 (0.09, 0.11)	3.65 (2.18, 5.31)
**40 to 54**	2470 (9.49)	1.28 (1.23, 1.34)	4.36 (2.61, 6.35)	2374 (9.11)	1.23 (1.18, 1.28)	4.47 (2, 8.4)
**55 to 69**	5884 (22.61)	4.12 (4.01, 4.22)	2.56 (1.79, 3.72)	4741 (18.21)	3.11 (3.02, 3.2)	3.15 (2.35, 4.17)
**70 to 84**	4498 (17.28)	6.74 (6.54, 6.94)	4.26 (2.85, 5.73)	3890 (14.94)	4.52 (4.38, 4.67)	3.62 (1.98, 5.64)
**+85**	626 (2.41)	4.98 (4.6, 5.38)	1.48 (0.07, 3.2)	787 (3.03)	3.03 (2.82, 3.25)	1.15 (-0.36, 2.86)

Abbreviations: NHW: Non-Hispanic White; NHB: Non-Hispanic Black; ASIR: Age-standardized incidence rate; CI: Confidence interval, AAPC: Average annual percent change; N/A: Not available.

#### Men

In men, 19,246 NEC cases were reported. The majority of them were among NHWs (71.99%) and individuals aged 55 to 69 years (42.81%). The highest ASIR was in the 70–84 age group (6.55 [6.38, 6.72]) and the greatest increase in ASIRs over 2000–2019 was among men below 39 years (AAPC: 4.45%; 2.46, 6.88) (Table 5).

Out of all the reported cases, 1,802 (9.36%) were among Hispanic individuals. The majority of cases were between 55 and 69 years (41.68%). The overall ASIR per 100,000 population for this group was 0.94 (0.89, 0.99). Cases between 70 and 84 years had the highest ASIR among all age groups (4.51 [4.09, 4.97]). Additionally, those aged 40–54 years had the highest rise with an AAPC of 4.08% (2.07, 6.96) (Table 5).

NHBs accounted for 15.19% of the cases of NEC in men. A majority of NHB cases occurred in individuals aged 55 to 69 years (45.04%). The overall ASIR per 100,000 population for NHBs was 2.27 (2.19, 2.36) and cases between 70 and 84 years had the highest ASIR among all age groups (10.87 [10.09, 11.69]). Those aged <39 years had the greatest AAPC (4.34% [1.36, 8.30]) (Table 5).

There were 13,855 (71.98%) reported NEC cases among NHWs. The majority of NHW cases occurred in individuals aged 55 to 69 years (42.46%). The overall ASIR per 100,000 population for NHW men was 1.43 (1.40, 1.45), with cases aged 70 to 84 years having the highest ASIR (6.74 [6.54, 6.94]). The greatest increase in the overall ASIR over the period of 2000 to 2019 occurred in those <39 years (AAPC: 4.59%; 2.72, 6.88) (Table 5).

#### Women

Between 2000 and 2019, a total of 17,555 cases of NEC were reported in women. The majority of them were among NHWs (69.36%) and 55–69 years (39.41%). The highest ASIR per 100,000 population occurred in those aged 70–84 years (4.48 [4.36, 4.61]). Furthermore, those below 39 years had the greatest increase in ASIRs over the period of 2000 to 2019 (AAPC: 4.88%; 3.27, 6.93) (Table 5).

Among all reported cases, 1,756 (10.00%) were among Hispanic individuals and the majority of cases were between 55 and 69 years (36.73%). The overall ASIR per 100,000 population for this group was 0.78 (0.74, 0.82). Individuals aged between 70 and 84 years had the highest ASIR (3.67 [3.35, 4.02]) (Table 5).

NHBs constituted 17.70% of the cases of NEC in women. A significant portion of NHB cases fell within the 55 to 69-year age group (42.58%). The overall ASIR per 100,000 population for NHB women was 1.80 (1.73, 1.86), with cases between 70 and 84 years having the highest ASIR among other age groups (7.25 [6.74, 7.78]). The greatest increase in ASIR from 2000–2019 occurred among those <39 years (AAPC: 6.90; 3.57, 11.74) (Table 5).

Among women with NEC, there were 12,177 (69.36%) reported cases of NHWs. The majority of NHW cases were in the 55 to 69 (38.93%) age group. The overall ASIR per 100,000 population for this group was 1.09 (1.07, 1.11), with cases between 70 and 84 years having the highest ASIR (4.52 [4.38, 4.67]). Furthermore, those between 40 and 54 years had the greatest increase in ASIRs over 2000–2019 (AAPC: 4.47%; 2.00, 8.40) (Table 5).

#### Age and sex patterns

The incidence rate of NEC increased with age and reached to the highest rate in 75–79 age group for both males and females. Also, the greatest numbers of incident cases were in males and females aged 65–69 years (S8 Fig).

### GIST

#### Overall incidence

Over 2000–2019, 6,572 cases of GIST were reported in all age groups in the US. The majority of the cases were men (54.21%), NHWs (67.10%), and aged between 55 and 69 years (38.99%). The ASIR per 100,000 population was 0.25 (0.24, 0.26) for men and 0.18 (0.18, 0.19) for women. The AAPCs for men and women were 2.80% (1.74, 4.02) and 4.95% (1.56, 8.10), respectively. NHW men had the highest ASIR (0.25 [0.24, 0.26]) and NHB women showed the greatest AAPC (6.59% [4.69, 9.04]) (Table 6, S9–S11 Figs).

**Table 6:** Counts and age-standardized rate of gastrointestinal stromal tumor incidence per 100,000 and average annual percent change from 2000 to 2019 in the United States, by age, sex, and race.

**All race/ethnicities**						
**Age group (years)**	**Men**			**Women**		
	**Case (%)**	**ASIR (95% CI)**	**AAPC (95% CI)**	**Case (%)**	**ASIR (95% CI)**	**AAPC (95% CI)**
**All**	3563 (54.21)	0.25 (0.24, 0.26)	2.80 (1.74, 4.02)	3009 (45.79)	0.18 (0.18, 0.19)	4.95 (1.56, 8.1)
**0 to 39**	264 (4.02)	0.03 (0.03, 0.04)	2.31 (0.13, 4.81)	247 (3.76)	0.03 (0.03, 0.04)	4.07 (1.49, 7.4)
**40 to 54**	842 (12.81)	0.27 (0.25, 0.29)	3.26 (1.07, 5.77)	684 (10.41)	0.22 (0.2, 0.23)	4.01 (2.52, 5.77)
**55 to 69**	1422 (21.64)	0.69 (0.65, 0.73)	2.5 (1.3, 3.97)	1140 (17.35)	0.5 (0.47, 0.53)	2.42 (1.07, 4.17)
**70 to 84**	917 (13.95)	1.03 (0.96, 1.1)	2.98 (1.36, 5.11)	803 (12.22)	0.68 (0.64, 0.73)	4.89 (2.07, 7.73)
**+85**	118 (1.80)	0.74 (0.61, 0.89)	-0.54 (-8.82, 13.13)	135 (2.05)	0.41 (0.35, 0.49)	N/A
**Hispanic**						
**Age groups**	**Men**			**Women**		
	**Case (%)**	**ASIR (95% CI)**	**AAPC (95% CI)**	**Case (%)**	**ASIR (95% CI)**	**AAPC (95% CI)**
**All**	490 (53.67)	0.23 (0.2, 0.25)	3.82 (2.07, 6.29)	423 (46.33)	0.17 (0.16, 0.19)	3.3 (1.55, 5.84)
**0 to 39**	69 (7.56)	0.03 (0.02, 0.04)	N/A	67 (7.34)	0.03 (0.03, 0.04)	N/A
**40 to 54**	152 (16.65)	0.26 (0.22, 0.3)	3.76 (0.11, 9.04)	123 (13.47)	0.21 (0.17, 0.25)	5.11 (1.47, 11.12)
**55 to 69**	187 (20.48)	0.67 (0.58, 0.78)	1.45 (-1.3, 5.45)	138 (15.12)	0.44 (0.37, 0.52)	5.63 (-1.1, 27.75)
**70 to 84**	73 (8.00)	0.79 (0.62, 0.99)	N/A	82 (8.98)	0.64 (0.51, 0.79)	N/A
**+85**	9 (0.99)	0.65 (0.3, 1.24)	N/A	13 (1.42)	0.51 (0.27, 0.87)	N/A
**NHB**						
**Age groups**	**Men**			**Women**		
	**Case (%)**	**ASIR (95% CI)**	**AAPC (95% CI)**	**Case (%)**	**ASIR (95% CI)**	**AAPC (95% CI)**
**All**	295 (53.44)	0.23 (0.2, 0.26)	4.14 (1.09, 8.62)	257 (46.56)	0.15 (0.13, 0.17)	6.59 (4.69, 9.04)
**0 to 39**	26 (4.71)	0.03 (0.02, 0.04)	N/A	12 (2.17)	0.01 (0.01, 0.02)	N/A
**40 to 54**	82 (14.86)	0.24 (0.19, 0.3)	N/A	62 (11.23)	0.16 (0.12, 0.21)	2.09 (-0.92, 5.5)
**55 to 69**	114 (20.65)	0.58 (0.48, 0.7)	N/A	111 (20.11)	0.45 (0.37, 0.55)	N/A
**70 to 84**	63 (11.41)	0.93 (0.71, 1.2)	N/A	65 (11.78)	0.61 (0.47, 0.77)	N/A
**+85**	10 (1.81)	1.05 (0.5, 1.93)	N/A	7 (1.27)	0.29 (0.12, 0.6)	N/A
**NHW**						
**Age groups**	**Men**			**Women**		
	**Case (%)**	**ASIR (95% CI)**	**AAPC (95% CI)**	**Case (%)**	**ASIR (95% CI)**	**AAPC (95% CI)**
**All**	2397 (54.35)	0.25 (0.24, 0.26)	5.1 (2.04, 7.79)	2013 (45.65)	0.19 (0.18, 0.2)	5.3 (1.06, 8.9)
**0 to 39**	132 (2.99)	0.03 (0.03, 0.04)	1.01 (-1.33, 3.44)	137 (3.11)	0.03 (0.03, 0.04)	4.65 (1.44, 8.59)
**40 to 54**	525 (11.90)	0.28 (0.26, 0.3)	7.81 (2.51, 13.92)	427 (9.68)	0.23 (0.21, 0.25)	3.88 (1.58, 6.49)
**55 to 69**	961 (21.79)	0.67 (0.63, 0.71)	2.94 (1.57, 4.68)	768 (17.41)	0.5 (0.47, 0.54)	2.09 (0.7, 3.78)
**70 to 84**	688 (15.60)	1.03 (0.95, 1.11)	2.9 (0.74, 5.75)	574 (13.02)	0.67 (0.61, 0.72)	4.81 (1.04, 8.16)
**+85**	91 (2.06)	0.72 (0.58, 0.89)	-1.29 (-9.92, 11.54)	107 (2.43)	0.41 (0.34, 0.5)	N/A

Abbreviations: NHW: Non-Hispanic White; NHB: Non-Hispanic Black; ASIR: Age-standardized incidence rate; CI: Confidence interval, AAPC: Average annual percent change; N/A: Not available.

#### Men

A total of 3,563 cases of GIST were reported in men during the period of 2000 to 2019. The majority of these cases were among NHWs (67.27%) and those aged 55 to 69 years (39.91%). The highest ASIR among all age groups belonged to those aged between 70 and 84 (1.03 [0.96, 1.10]) and the greatest increase in ASIR over 2000–2019 was among men between 40 and 54 years (AAPC: 3.26%; 1.07, 5.77) (Table 6).

Out of all the reported cases, 490 (13.75%) were among Hispanic individuals. The majority of cases were between 55 and 69 years (38.16%). The overall ASIR per 100,000 population for this group was 0.23 (0.20, 0.25). Cases between 70 and 84 years had the highest ASIR (0.79 [0.62, 0.99]) (Table 6).

NHBs accounted for 8.28% of the cases of NEC in men. A majority of NHB cases occurred in individuals aged 55 to 69 years (38.64%). The overall ASIR per 100,000 population for NHBs was 0.23 (0.20, 0.26), and cases above 85 years had the highest ASIR among all age groups (1.05 [0.50, 1.93]). NHB men showed an overall significant AAPC of 4.14% (1.09, 8.62) (Table 6).

There were 2,397 (67.27%) reported NEC cases among NHWs. The majority of NHW cases occurred in individuals aged 55 to 69 years (40.09%). The overall ASIR per 100,000 population for NHW men was 0.25 (0.24, 0.26), with cases aged 70 to 84 years having the highest ASIR (1.03 [0.95, 1.11]). The greatest increase in ASIR over the period of 2000 to 2019 occurred in those between 40 and 54 years (AAPC: 7.81%; 2.51, 13.92) (Table 6).

#### Women

Between 2000 and 2019, a total of 3,009 cases of GIST were reported in women. The majority of these cases were among NHWs (66.90%) and most of them were in the 55 to 69 age group (37.89%). The highest ASIR per 100,000 population occurred in those aged 70 to 84 (0.68 [0.64, 0.73]).

Among all reported cases, 423 (14.06%) were among Hispanic individuals and the majority of cases were between 55 and 69 years (32.62%). The overall ASIR per 100,000 population for this group was 0.17 (0.16, 0.19). Individuals aged 70 to 84 years had the highest ASIR compared to other age groups, with ASIR of 0.64 (0.51, 0.79) (Table 6).

NHBs constituted 8.54% of the cases of GIST in women. The greatest ratio of NHB cases were in the 55 to 69-year age group (43.19%). The overall ASIR per 100,000 population for NHB women was 0.15 (0.13, 0.17) (Table 6).

Among women with GIST, there were 2,013 (66.90%) reported cases of NHWs. The majority of NHW cases were in the 55 to 69 age group (38.15%). The overall ASIR per 100,000 population for this group was 0.19 (0.18, 0.20), with cases between 70 and 84 years having the highest ASIR (0.67 [0.61, 0.72]) (Table 6).

#### Age and sex patterns

The highest incidence rate of GIST in both men and women was in the 75–79 age group. Also, the incident cases increased with advancing age up to 55–59 age group for females and 60–64 age group for males, before decreasing then after (S12 Fig).

## Discussion

This study provided an investigation of small intestine cancer incidence trends in the US over 21 years from 2000 to 2020. We analyzed the incidence trends, considering the sex differences, age groups, racial disparities, and histopathological subtypes of the cancer. Moreover, we addressed the potential impact of the COVID-19 pandemic on the incidence rates. Generally, the findings indicate an overall increasing incidence of small intestine cancer in both sexes and across diverse races/ethnicities from 2000 to 2019. However, with the outbreak of the COVID-19 pandemic, a notable decline in reported cases was observed in 2020 compared to the preceding year.

Analysis of the overall incidence rates of intestinal cancer between 2000 and 2019 demonstrated an increasing rate in both men and women and across all races/ethnicities, with men consistently having a higher ASIR compared to women. However, despite NHWs constituting the majority of small intestine cancers, NHBs demonstrated the highest incidence rates across 2000–2019 in both men and women. Notably, rates were increasing at a higher pace in NHB women compared to NHB men, with NHB women demonstrating the highest ASIR and AAPC among all races, suggesting that these individuals must be taken into account as a potentially high-risk group for small intestine cancer incidence. Analyzing the age groups, we observed increasing rates of small intestine cancer incidence in almost all age groups, except for men above 85 years, which showed consistent rates from 2000 to 2019. In both sexes, the highest incidence rates were observed in the 70 to 84-year-old population. Generally, the overall observed trends of small intestine cancer from 2000 to 2019 are comparable with previous reports in the US. A recent study on the incidence of small intestine cancers in the US between 1976 and 2016 demonstrated that the incidences doubled from 12.1 to 27.9 per million [29]. A previous study using the SEER database from 1992 to 2006 also showed an incidence rate of 2.1/100,000 persons-years, with higher rates in men compared to women. Moreover, as in our study, black races exhibited the highest incidence rates [30]. Findings from another study utilizing the SEER data also showed an increase in incidence rates from 11.8 cases per million in 1973 to 22.7 cases per million in 2004, with a male predominance observed across all histopathological types [13]. Similarly, another analysis of population-based cancer registries in the US from 1995 to 2008 showed higher incidence rates in men while also showing that the rates were 42% higher among black men than white men, and 46% higher in black women compared to white women [31]. The overall rise in the incidence of small intestine cancer is often attributed to enhanced diagnosis of the cancer due to increased utilization of diagnostic measures with improved access to the intestine. Generally, the small intestine presents a considerable challenge for diagnostic inspection due to its limited accessibility, which poses difficulties for both radiologic and endoscopic examinations. However, technological advancements, including functional imaging, capsule endoscopy, and small-bowel enteroscopy, are progressively improving accessibility for the diagnosis of small intestine tumors. Nevertheless, consideration must be given to changes in risk factors for small intestine cancer, encompassing factors such as smoking, alcohol consumption, dietary habits, as well as the presence of conditions like celiac disease and Crohn’s disease. Understanding how these factors vary across age and racial categories is essential for gaining additional insights into the reasons behind the observed increase in small intestine cancer incidence [32].

Our study also encompassed distinct analyses of various histopathological subtypes of small cancer intestine. Among the histopathological subtypes, NEC was the category with the highest incident cases, constituting 54.26% of all reported cases and showing the highest incidence rates between 2000 and 2019. The general incidence patterns of NEC were similar to what was observed in the overall incidence of small intestine cancer. Accordingly, an increasing rate of NEC was observed in both sexes and across all races/ethnicities, which showed the highest incidence rates in 70 to 84-year-old populations. Moreover, individuals above 85 years showed a steady incidence trend of NEC over 2000–2019, while in other age groups, an increasing rate was observed. Similar to what was observed in total incidence trends, NHBs demonstrated the highest incidence rates of NEC among all races. Generally, NECs arise from the diffuse system of neuroendocrine cells, which are most frequently seen in the gastrointestinal and bronchopulmonary systems and generally demonstrate a slow-growing pattern [33]. An analysis of the SEER database between 2000 and 2012 suggested that among all neuroendocrine tumors, the small intestine neuroendocrine tumors exhibited the second highest incidence rates after the lung [34]. A more recent investigation of gastrointestinal neuroendocrine tumors between 1977 and 2016 showed an increasing incidence of these tumors over the four decades, with incidence rates reaching 4 per 100,000 populations in 2016. Notably, among all gastrointestinal sites, small intestine showed the highest incidence rates in all decades [35]. In another study on the SEER data from 2000 to 2017, small intestine neuroendocrine tumors had the highest prevalence compared to colon and rectum, with the highest cases being males and white races [36]. In line with our observations, NEC constituted the most common histopathological subtype with the highest incidence rates between 1992 and 2006 in another previous US population-based study [30]. Moreover, black races exhibited higher incidence rates of NEC compared to other races [30]. Another study on neuroendocrine tumors of the gastrointestinal tract in the US between 1975 and 2008 reported that these tumors were more prevalent in the small intestine compared to other parts of the gastrointestinal tract, including the rectum, colon, stomach, and appendix. Notably, the incidence rates significantly increased through 1975–2008 for the neuroendocrine tumors of all parts of the gastrointestinal tract, except for the appendix [37]. Generally, our results confirm previous investigations on NEC being a common histopathological type of small intestine cancer with increasing rates over the years, which is most possibly due to increasing utilization of diagnostic modalities. The increasing rates of small intestine cancer and NEC in particular among black races warrant further attention to these individuals.

On the other hand, we observed distinct trends of adenocarcinoma as the second most common histopathological subtype reported over 2000–2019. Generally, findings showed an increasing incidence rates of adenocarcinoma in men but not women over the study period. Notably, our findings demonstrated declining incidence rates of adenocarcinoma, particularly between 2017 and 2019, which was a distinct pattern in contrast to other histopathological subtypes. However, narrowing the analysis into age groups, we observed increasing trends in women under 39 years old, with the highest AAPC reported in NHB women of this age group. Similar to the total incidence rates and what was observed in the NEC subtype, the highest incidence rates of adenocarcinoma were also observed in NHBs. Small intestine adenocarcinoma often occurs in the glandular epithelium, and due to the lack of specific symptoms and non-accessible tumor sites, they are often diagnosed at later stages. Therefore, the outcomes are on average worse than for other related malignancies [38]. In our findings, we observed that unlike NEC, the highest incidence rates of small intestine adenocarcinoma were observed in ages above 85 in both men and women, suggesting that this histological subtype was generally diagnosed at higher ages, which might contribute to worse survival. A US population-based study using the national cancer database between 1985 and 2005 also showed that adenocarcinomas of the small intestine had the lowest survival rates compared to other histopathological types [13]. Moreover, in a SEER-based study between 2004 and 2015 among cases aged 65 or older, it was observed that the 3-year and 5-year overall survival was 36.0% and 26.5%, respectively [39]. Another study in the same period also reported a 3-year and 5-year overall survival of 39.3% and 31.8% among small intestine adenocarcinoma regardless of age, respectively [40]. Considering the incidence patterns, a study using the SEER data over 1973–2004 also showed an increasing incidence of small intestine adenocarcinoma with an APC of 1.3% [13]. Notably, the same study, utilized the data from the national cancer database between 1985 and 2005 and demonstrated that adenocarcinomas were the most prevalent subtype of small intestine malignancies till 2000, however, these tumors were surpassed by carcinoid tumors–a subtype of NEC—since then [13]. Our findings confirm these trends after 2000, as we reported that adenocarcinomas were the second most common histologic subtype after NECs. Notably, we also demonstrated a changing pattern with decreasing incidence rates of adenocarcinoma, particularly among women, between 2017 and 2019, which requires further prospective navigation through subsequent years to ascertain if this trend persists.

Our findings finally demonstrated that the incidence pattern of small intestine GIST, the third common histopathological subtype, also followed the general patterns observed for NEC. The incidence rates of GIST increased in both sexes over 2000–2019, with the highest incidence rates observed in 70–84 age groups. However, despite the two previous histopathological subtypes, the highest incidence rates were observed in NHWs, while NHBs still showed the highest AAPCs during the study period. Generally, GIST is the most commonly reported mesenchymal tumor of the gastrointestinal tract originating from interstitial cells of Cajal or the stem cell precursors. A recent SEER-based study on the GIST epidemiology between 2010 and 2019 also demonstrated an increasing ASIR for all GISTs of the gastrointestinal tract from 0.79 per 100,000 person-years in 2010 to 1.02 per 100,000 person-years in 2019 [41]. Moreover, small intestine GIST was reported to be the major site of GIST occurrence after the stomach. However, unlike the increasing incidence of stomach GIST, no significant increase in small intestine GIST was reported between 2010 and 2019, while an increasing trend for localized stage small intestine GIST was observed [41]. Another study utilizing the data from 2002 to 2012 also reported an increasing incidence of small intestine GIST in patients above 50 years, which was less pronounced than the increase in stomach GIST [42]. Generally, although small intestine GIST represents lower incidence rates compared to other histological types, our findings still highlighted increasing rates across both sexes between 2000 and 2019. Notably, we found a significant APC between 2000 and 2002, suggesting a remarkable increase in GIST incidence within this period. This observation is most likely due to the discovery of c-Kit (CD117), which facilitated the diagnosis of GIST, that was largely misclassified as smooth muscle tumors before the early 2000s [42, 43].

To examine the influence of the COVID-19 pandemic on the incidence of small intestine cancer, we analyzed the alterations in incidence rates in 2020 relative to the preceding year. The results unveiled a reduction in the ASIR of small intestine cancer across all racial and sex categories subsequent to the onset of the pandemic. The reduced incidence of small intestine cancer during the COVID-19 pandemic, as reported in previous studies on various cancer types [44–46], may be ascribed to impaired screening and reduced cancer diagnostic measures during the pandemic. Accordingly, a recent study has identified a notable decrease in the screening and diagnosis of several cancers following the outbreak of the pandemic [47]. Notably, findings of a meta-analysis reported an overall decline of 37.3% for diagnostic tests and a 27.0% decrease for cancer diagnosis during the pandemic [48]. The strain on medical centers due to the surge in COVID-19 cases, coupled with patients’ hesitancy in seeking diagnostic procedures, could have led to the underreporting of cancer cases. The delayed diagnosis and postponement of treatment during the pandemic may also have enduring implications for the survival outcomes of small intestine cancer in the ensuing years. Overall, it is plausible that the COVID-19 pandemic, with its pervasive impact on healthcare delivery, has affected the epidemiology of cancers and the burden experienced by individuals with cancer. The consequences of delayed diagnosis of small intestine cancer and its effects on patient survival necessitate additional research.

There are several strengths to this study. This study stands as the initial exploration of the updated SEER database to comprehensively assess the incidence trends of small intestine cancer based on several key demographic factors. Besides analyzing the overall trends in cancer incidence, we conducted subgroup analyses on various pathological subtypes, including NECs, adenocarcinoma, and GIST to enhance the understanding of the incidence rates within different pathologic classifications. Considering the substantial impact of the COVID-19 pandemic on cancer diagnosis, our study also furnished an overview of the alterations in small intestine cancer incidence rates during the initial year of the pandemic compared to the preceding year. However, our findings might be influenced by some limitations. First, delay adjustment was not possible for the calculation of the small intestine cancer incidence rates, which might have resulted in the underestimation of the rates, particularly within the recent years of the study period [49]. In order to develop a reliable delay-adjustment model, a substantial number of cases must be available in the submissions to analyze the delay pattern for specific cancer sites or racial groups, while the data was not available for small intestine cancer [50]. Such an underestimation might affect the interpretation of trends, especially when assessing the impact of the COVID-19 pandemic. Registries generally permit a fixed period for reporting diagnosed cases before releasing initial case counts for a specific diagnosis year. However, additional cases are often reported after these initial counts, leading to more complete data in subsequent releases. This results in earlier releases underestimating the true incidence rates. Since reporting delays cannot exceed the duration that the registry has been collecting data, these methods typically estimate a truncated delay distribution. These methods are particularly useful for groups of registries that started collecting data in the same year, helping to adjust incidence rates more accurately for reporting delays [51]. Furthermore, an inherent limitation of the SEER program database lies in the potential for misclassification bias stemming from the collection of demographic data, including race and ethnicity, from diverse sources such as administrative databases, patient intake, or provider notes. This bias, if present, might influence the observed disparities in incidence rates among racial and ethnic groups. Misclassification or inaccuracies in racial and ethnic data can lead to either an underestimation or overestimation of incidence rates in specific groups. This potential bias underscores the importance of careful interpretation of the trends and disparities observed in cancer incidence rates, and highlights the need for improved accuracy in demographic data collection. A systematic review showed that the quality issues of data vary by variable and can lead to specific analytical errors. Social determinants of health data problems are usually not random, with Hispanic patients’ data being more susceptible to plausibility and misclassification errors compared to other racial/ethnic groups [52]. Another study revealed an overall 11% disagreement between registry and interview race/ethnicity data, which were linked to factors such as being non-White, younger age, marital status, and being foreign-born but preferring English [53]. Moreover, the limited sample size precluded sub-grouping our analysis according to race and ethnicity groups, specifically American Indian/Alaskan Native, Asian/Pacific Islander, and Native Hawaiian. Consequently, our findings may not comprehensively capture the characteristics of individuals from these specific racial and ethnic backgrounds. Subgroup analyses within the SEER database can provide insights into cancer epidemiology by age, sex, and race/ethnicity, but they have some limitations that should be considered. Smaller sample sizes in subgroups can reduce statistical power and reliability. Confounding variables, if not properly controlled, may bias the results, and significant findings in subgroups might occur by chance. Missing data, if not addressed effectively, can introduce bias in clinical research, particularly when data are missing not at random. It can affect both validity and reliability of the findings. This influence can be in several ways, including bias altering sample characteristics, reduced statistical power due to smaller sample sizes and increased variability, and limitations on generalizability. To resolve this, strategies such as imputation techniques, sensitivity analyses, and appropriate statistical methods selection are used. Addressing missing data necessitates careful consideration of study design, reasons for missing data, and potential biases [54]. In the SEER study, the cases with unknown age or non-binary sex have been excluded from the database [55]. Therefore, the missing information were not included in the analysis. Also, the SEER*Stat using data of SEER use multiple techniques to handle missing data and reduce bias.

### Clinical research relevance

The results of this study, which demonstrate that the incidence of small intestine cancer has increased over the previous 20 years, highlight the significance of the increasing burden of this cancer in the US. Notably, greater incidence rates among NHB individuals emphasize the necessity for focused clinical investigation, especially for NHB women. These patterns suggest that NHB women should be viewed as a potentially high-risk population that requires targeted screening and early detection initiatives. Therefore, in order to promote equitable healthcare delivery, clinicians should be aware of these demographic inequalities and take them into account when formulating diagnostic and treatment strategies. Moreover, considering the complicated access to the small intestine, which results in delayed diagnosis of some cancer subtypes, more efforts should be focused on the development of more feasible screening strategies. Additionally, the COVID-19 pandemic’s effects on cancer diagnosis and reporting present a chance to research the ways in which external factors affect cancer epidemiology.

### Basic research relevance

The observed racial and age-related disparities in small intestine cancer incidence provide a valuable basis for basic research into the genetic and environmental factors contributing to these differences. Particularly, future research could investigate the molecular mechanisms underlying the higher susceptibility in NHB populations and the distinct age-related patterns in cancer incidence. Understanding these mechanisms could lead to the development of more effective prevention strategies and personalized treatments.

### Implications for decision-makers

For healthcare policymakers, the decline in cancer diagnoses during the COVID-19 pandemic suggests the need for robust healthcare systems that can maintain essential services during crises. Moreover, there is a clear need for policies that promote equitable access to cancer screening and treatment services, especially for high-risk groups. Policymakers should consider allocating resources towards public health initiatives that raise awareness about small intestine cancer and encourage regular screenings.

### Implications for future studies

Longitudinal studies tracking the future incidence trends will be crucial in understanding the long-term impact of the COVID-19 pandemic on cancer diagnosis and reporting. Moreover, researchers should also consider investigating the role of socioeconomic factors and healthcare access in contributing to the disparities identified in this study. Expanding the geographic scope of the research to include international data could provide a more comprehensive understanding of small intestine cancer trends and inform global health strategies.

## Conclusions

Findings indicated an increasing incidence rate of small intestine cancer across both sexes and all races/ethnicities. Although the occurrence of the cancer was generally higher in white races, the higher incidence rates and AAPCs in the black race warrant attention to this particular population group. Our findings also showed a notable decline in the reported cases of small intestine cancer after the COVID-19 pandemic, which might be due to the impaired diagnostic measures during the pandemic. The consequences of cancer underdiagnosis and its impact on the survival of patients requires further research.

## Supporting information

S1 Table Counts and age-standardized rate of small intestine cancer incidence per 100,000 and average annual percent change from 2015 to 2019 in the United States, by age, sex, and race. (DOCX)

S2 Table Results of the tests of parallelism for small intestine cancer incidence rate over 2000–2019 in the United States. (DOCX)

S3 Table Identical trends of small intestine cancer incidence rate over 2000–2019 in the United States. (DOCX)

S1 Fig The age-adjusted incidence rate of adenocarcinoma per 100,000 people over 2000–2019 and in 2020 in the United States, by sex. APC: annual percent change. * Represent p-value less than 0.05.
(DOCX)

S2 Fig The age-adjusted incidence rate of adenocarcinoma over 2000–2019 and in 2020 in the United States, by race/ethnicity. APC: annual percent change. * Represent p-value less than 0.05.
(DOCX)

S3 Fig The age-adjusted incidence rate of adenocarcinoma over 2000–2019 and in 2020 in the United States, by age. APC: annual percent change. * Represent p-value less than 0.05.
(DOCX)

S4 Fig Incident numbers and incidence rate (per 100,000 population) of adenocarcinoma in the United States among males and females in each age group. (DOCX)

S5 Fig The age-adjusted incidence rate of neuroendocrine carcinoma per 100,000 people over 2000–2019 and in 2020 in the United States, by sex. APC: annual percent change. * Represent p-value less than 0.05.
(DOCX)

S6 Fig The age-adjusted incidence rate of neuroendocrine carcinoma over 2000–2019 and in 2020 in the United States, by race/ethnicity. APC: annual percent change. * Represent p-value less than 0.05.
(DOCX)

S7 Fig The age-adjusted incidence rate of neuroendocrine carcinoma over 2000–2019 and in 2020 in the United States, by age. APC: annual percent change. * Represent p-value less than 0.05.
(DOCX)

S8 Fig Incident numbers and incidence rate (per 100,000 population) of neuroendocrine carcinoma in the United States among males and females in each age group. (DOCX)

S9 Fig The age-adjusted incidence rate of gastrointestinal stromal tumor per 100,000 people over 2000–2019 and in 2020 in the United States, by sex. APC: annual percent change. * Represent p-value less than 0.05.
(DOCX)

S10 Fig Age-adjusted incidence rate of gastrointestinal stromal tumor over 2000–2019 and in 2020 in the United States, by race/ethnicity. APC: annual percent change. * Represent p-value less than 0.05.
(DOCX)

S11 Fig Age-adjusted incidence rate of gastrointestinal stromal tumor over 2000–2019 and in 2020 in the United States, by age. APC: annual percent change. * Represent p-value less than 0.05.
(DOCX)

S12 Fig Incident numbers and incidence rate (per 100,000 population) of gastrointestinal stromal tumor in the United States among males and females in each age group. (DOCX)

S1 Graphical abstract (TIF)
