# Supplementary material for: A population-based study on incidence trends of small intestine cancer in the United States from 2000 to 2020
Source: PLoS One. 2024 Aug 19;19(8):e0307019. doi: 10.1371/journal.pone.0307019 (PMC11332941; doi:10.1371/journal.pone.0307019)
Supplement: S1 Table — (DOCX) [file pone.0307019.s001.docx]

**S1 Table.** Counts and age-standardized rate of small intestine cancer incidence per 100,000 and average annual percent change from 2015 to 2019 in the United States, by age, sex, and race.

| **All race/ethnicities** | | | | | | |
| --- | --- | --- | --- | --- | --- | --- |
| **Age group (years)** | **Men** | | | **Women** | | |
|  | **Case (%)** | **ASIR (95% CI)** | **AAPC(95% CI)** | **Case (%)** | **ASIR (95% CI)** | **AAPC(95% CI)** |
| **All** | 11758(53.59) | 2.91 (2.86, 2.97) | 1.32 (-0.69, 1.72) | 10181(46.41) | 2.17 (2.12, 2.21) | 0.94 (-0.3, 1.6) |
| **0 to 39** | 424 (1.93) | 0.21 (0.19, 0.23) | 2.22 (1.19, 3.37) | 452 (2.06) | 0.22 (0.2, 0.24) | 3.63 (2.75, 4.68) |
| **40 to 54** | 1991 (9.08) | 2.56 (2.45, 2.68) | 2.54 (1.87, 3.29) | 1752 (7.99) | 2.21 (2.11, 2.32) | 0.73 (-4.15, 3.56) |
| **55 to 69** | 4766 (21.72) | 7.44 (7.23, 7.65) | 0.29 (-2.81, 1.21) | 3930(17.91) | 5.61 (5.43, 5.79) | 0.4 (-1.95, 1.42) |
| **70 to 84** | 3913(17.84) | 14.78 (14.31, 15.25) | 1.92 (1.55, 2.33) | 3260(14.86) | 9.76 (9.43, 10.1) | 1.09 (-0.38, 1.62) |
| **+85** | 664 (3.03) | 13.05 (12.07, 14.08) | -1.69 (-5.96, 0.37) | 787 (3.59) | 8.35 (7.77, 8.95) | -0.23 (-0.9, 0.33) |
| **Hispanic** | | | | | | |
| **Age groups** | **Men** | | | **Women** | | |
|  | **Case (%)** | **ASIR (95% CI)** | **AAPC(95% CI)** | **Case (%)** | **ASIR (95% CI)** | **AAPC(95% CI)** |
| **All** | 1430 (53.76) | 2.26 (2.13, 2.38) | 2 (1.28, 2.97) | 1230 (46.24) | 1.66 (1.56, 1.75) | 0.44 (-2.52, 1.38) |
| **0 to 39** | 93 (3.50) | 0.16 (0.13, 0.19) | 2.59 (0.35, 5.36) | 94 (3.53) | 0.16 (0.13, 0.2) | 2.95 (0.48, 6.22) |
| **40 to 54** | 354 (13.31) | 1.92 (1.72, 2.13) | 3.11 (1.82, 4.84) | 274 (10.30) | 1.5 (1.33, 1.69) | 2.56 (1.17, 4.34) |
| **55 to 69** | 563 (21.17) | 5.8 (5.32, 6.3) | 1.5 (0.23, 3.25) | 447 (16.80) | 4.17 (3.79, 4.57) | 2.12 (0.88, 3.75) |
| **70 to 84** | 357 (13.42) | 11.33 (10.17, 12.58) | 2.28 (0.76, 4.29) | 343 (12.89) | 8.03 (7.2, 8.93) | -5.38 (-11.78, 0.66) |
| **+85** | 63 (2.37) | 11.49 (8.83, 14.71) | -0.41 (-2.9, 3.07) | 72 (2.71) | 7.39 (5.78, 9.3) | -2.1 (-4.21, 0.46) |
| **NHB** | | | | | | |
| **Age groups** | **Men** | | | **Women** | | |
|  | **Case (%)** | **ASIR (95% CI)** | **AAPC(95% CI)** | **Case (%)** | **ASIR (95% CI)** | **AAPC(95% CI)** |
| **All** | 1831 (49.04) | 4.75 (4.52, 4.99) | 1.97 (1.22, 2.9) | 1903 (50.96) | 3.74 (3.57, 3.91) | 2.31 (-1.62, 3.91) |
| **0 to 39** | 70 (1.88) | 70 (1.87) | 1.79 (-1.1, 5.1) | 103 (2.76) | 0.41 (0.33, 0.49) | 5.13 (3.12, 7.77) |
| **40 to 54** | 373 (9.99) | 373 (9.99) | 2.16 (0.46, 4.11) | 422 (11.30) | 4.25 (3.85, 4.68) | -2.34 (-9.22, 3.24) |
| **55 to 69** | 816 (21.86) | 816 (21.85) | 1.71 (0.7, 2.98) | 792 (21.21) | 10.03 (9.34, 10.75) | 0.93 (-5.17, 3.07) |
| **70 to 84** | 498 (13.34) | 498 (13.34) | 2.3 (1.09, 3.85) | 500 (13.39) | 15.52 (14.18, 16.95) | 1.29 (-4.13, 2.6) |
| **+85** | 74 (1.98) | 74 (1.98) | 0.56 (-2.59, 4.86) | 86 (2.30) | 11.49 (9.19, 14.19) | -3.04 (-20.76, 0.6) |
| **NHW** | | | | | | |
| **Age groups** | **Men** | | | **Women** | | |
|  | **Case (%)** | **ASIR (95% CI)** | **AAPC(95% CI)** | **Case (%)** | **ASIR (95% CI)** | **AAPC(95% CI)** |
| **All** | 7817 (54.51) | 2.95 (2.88, 3.02) | 1.29 (0.48, 1.73) | 6523 (45.49) | 2.17 (2.12, 2.23) | 0.84 (-0.38, 1.51) |
| **0 to 39** | 223 (1.56) | 0.22 (0.2, 0.26) | 2.56 (1.1, 4.14) | 208 (1.45) | 0.21 (0.19, 0.25) | 2.79 (1.69, 3.98) |
| **40 to 54** | 1124 (7.84) | 2.64 (2.49, 2.8) | 1.73 (-1.48, 2.69) | 960 (6.69) | 2.27 (2.12, 2.42) | -1.08 (-6.26, 2.67) |
| **55 to 69** | 3138 (21.88) | 7.4 (7.14, 7.66) | 0.39 (-3.65, 1.29) | 2503 (17.45) | 5.58 (5.36, 5.8) | 0.58 (-0.91, 1.39) |
| **70 to 84** | 2840 (19.80) | 14.98 (14.43, 15.55) | 1.99 (1.52, 2.5) | 2255 (15.73) | 9.76 (9.36, 10.18) | 1.81 (1.37, 2.29) |
| **+85** | 492 (3.43) | 12.96 (11.84, 14.16) | -2.42 (-7.53, 0.38) | 597 (4.16) | 8.55 (7.87, 9.26) | 0.39 (-1.04, 1.18) |

Abbreviations: NHW: Non-Hispanic White; NHB: Non-Hispanic Black; ASIR: Age-standardized incidence rate; CI: Confidence interval, AAPC: Average annual percent change.
