# Supplementary material for: A population-based study on incidence trends of small intestine cancer in the United States from 2000 to 2020
Source: PLoS One. 2024 Aug 19;19(8):e0307019. doi: 10.1371/journal.pone.0307019 (PMC11332941; doi:10.1371/journal.pone.0307019)
Supplement: S2 Table — (DOCX) [file pone.0307019.s002.docx]

**S2 Table.** Results of the tests of parallelism for small intestine cancer incidence rate over 2000-2019 in the United States.

| **Race** | **Sex** | **Age** | **Subtype** | **Race** | **Sex** | **Age** | **Subtype** | **P Value** |
| --- | --- | --- | --- | --- | --- | --- | --- | --- |
| **Cohort 1** | | | | **Cohort 2** | | | |  |
| All | Female | All | All | All | Male | All | GIST | 0.16 |
| Hispanic | Female | All | GIST | Hispanic | Female | All | NEC | 0.65 |
| NHB | Female | All | GIST | NHB | Female | All | NEC | 0.16 |
| NHW | Female | All | All | NHW | Female | All | GIST | 0.19 |
| NHW | Female | All | GIST | NHW | Female | All | NEC | 0.06 |
| All | Male | All | GIST | All | Male | All | NEC | 0.62 |
| Hispanic | Male | All | Adenocarcinoma | Hispanic | Male | All | All | 0.06 |
| Hispanic | Male | All | GIST | Hispanic | Male | All | GIST | 0.14 |
| NHB | Male | All | All | NHB | Male | All | GIST | 0.05 |
| NHB | Male | All | GIST | NHB | Male | All | NEC | 0.44 |
| NHW | Male | All | All | NHW | Male | All | GIST | 0.09 |
| NHW | Male | All | GIST | NHW | Male | All | NEC | 0.55 |
| All | Both | All | GIST | All | Both | All | NEC | 0.17 |
| Hispanic | Both | All | GIST | Hispanic | Both | All | NEC | 0.08 |
| Hispanic | Both | All | GIST | Hispanic | Both | All | NEC | 0.8 |
| NHB | Both | All | All | NHB | Both | All | GIST | 0.05 |
| NHB | Both | All | GIST | NHB | Both | All | NEC | 0.48 |
| NHW | Both | All | All | NHW | Both | All | GIST | 0.06 |
| NHW | Both | All | GIST | NHW | Both | All | NEC | 0.05 |
| All | Female | All | Adenocarcinoma | NHB | Female | All | Adenocarcinoma | 0.39 |
| NHB | Female | All | Adenocarcinoma | NHW | Female | All | Adenocarcinoma | 0.76 |
| NHB | Female | All | Adenocarcinoma | NHW | Female | All | Adenocarcinoma | 0.39 |
| All | Female | All | All | Hispanic | Female | All | All | 0.24 |
| All | Female | All | All | NHB | Female | All | All | 0.10 |
| All | Female | All | All | NHW | Female | All | All | 0.43 |
| Hispanic | Female | All | All | NHW | Female | All | All | 0.18 |
| NHB | Female | All | All | NHW | Female | All | All | 0.38 |
| All | Female | All | GIST | Hispanic | Female | All | GIST | 0.91 |
| All | Female | All | GIST | NHB | Female | All | GIST | 0.09 |
| All | Female | All | GIST | NHW | Female | All | GIST | 0.67 |
| Hispanic | Female | All | GIST | NHB | Female | All | GIST | 0.17 |
| Hispanic | Female | All | GIST | NHW | Female | All | GIST | 0.59 |
| NHB | Female | All | GIST | NHW | Female | All | GIST | 0.05 |
| All | Female | All | NEC | Hispanic | Female | All | NEC | 0.36 |
| All | Female | All | NEC | NHB | Female | All | NEC | 0.14 |
| All | Female | All | NEC | NHW | Female | All | NEC | 0.41 |
| Hispanic | Female | All | NEC | NHB | Female | All | NEC | 0.92 |
| Hispanic | Female | All | NEC | NHW | Female | All | NEC | 0.73 |
| NHB | Female | All | NEC | NHW | Female | All | NEC | 0.32 |
| All | Male | All | Adenocarcinoma | NHB | Male | All | Adenocarcinoma | 0.84 |
| All | Male | All | Adenocarcinoma | NHW | Male | All | Adenocarcinoma | 0.31 |
| Hispanic | Male | All | Adenocarcinoma | NHB | Male | All | Adenocarcinoma | 0.47 |
| Hispanic | Male | All | Adenocarcinoma | NHW | Male | All | Adenocarcinoma | 0.06 |
| NHB | Male | All | Adenocarcinoma | NHW | Male | All | Adenocarcinoma | 0.63 |
| All | Male | All | All | Hispanic | Male | All | All | 0.41 |
| All | Male | All | All | NHB | Male | All | All | 0.18 |
| All | Male | All | All | NHW | Male | All | All | 0.06 |
| Hispanic | Male | All | All | NHB | Male | All | All | 0.93 |
| Hispanic | Male | All | All | NHW | Male | All | All | 0.42 |
| NHB | Male | All | All | NHW | Male | All | All | 0.18 |
| All | Male | All | GIST | Hispanic | Male | All | GIST | 0.38 |
| All | Male | All | GIST | NHB | Male | All | GIST | 0.65 |
| All | Male | All | GIST | NHW | Male | All | GIST | 0.52 |
| Hispanic | Male | All | GIST | NHB | Male | All | GIST | 0.83 |
| Hispanic | Male | All | GIST | NHW | Male | All | GIST | 0.30 |
| NHB | Male | All | GIST | NHW | Male | All | GIST | 0.57 |
| All | Male | All | NEC | Hispanic | Male | All | NEC | 0.55 |
| All | Male | All | NEC | NHW | Male | All | NEC | 0.46 |
| Hispanic | Male | All | NEC | NHB | Male | All | NEC | 0.71 |
| Hispanic | Male | All | NEC | NHW | Male | All | NEC | 0.68 |
| NHB | Male | All | NEC | NHW | Male | All | NEC | 0.12 |
| All | Both | All | Adenocarcinoma | Hispanic | Both | All | Adenocarcinoma | 0.41 |
| All | Both | All | Adenocarcinoma | NHB | Both | All | Adenocarcinoma | 0.32 |
| All | Both | All | Adenocarcinoma | NHW | Both | All | Adenocarcinoma | 0.43 |
| Hispanic | Both | All | Adenocarcinoma | NHB | Both | All | Adenocarcinoma | 0.08 |
| Hispanic | Both | All | Adenocarcinoma | NHW | Both | All | Adenocarcinoma | 0.28 |
| NHB | Both | All | Adenocarcinoma | NHW | Both | All | Adenocarcinoma | 0.23 |
| All | Both | All | All | Hispanic | Both | All | All | 0.96 |
| All | Both | All | All | NHB | Both | All | All | 0.20 |
| All | Both | All | All | NHW | Both | All | All | 0.16 |
| Hispanic | Both | All | All | NHB | Both | All | All | 0.77 |
| Hispanic | Both | All | All | NHW | Both | All | All | 0.87 |
| NHB | Both | All | All | NHW | Both | All | All | 0.38 |
| All | Both | All | GIST | Hispanic | Both | All | GIST | 0.53 |
| All | Both | All | GIST | NHB | Both | All | GIST | 0.23 |
| All | Both | All | GIST | NHW | Both | All | GIST | 0.36 |
| Hispanic | Both | All | GIST | NHB | Both | All | GIST | 0.52 |
| Hispanic | Both | All | GIST | NHW | Both | All | GIST | 0.33 |
| NHB | Both | All | GIST | NHW | Both | All | GIST | 0.17 |
| All | Both | All | NEC | Hispanic | Both | All | NEC | 0.62 |
| All | Both | All | NEC | NHW | Both | All | NEC | 0.30 |
| Hispanic | Both | All | NEC | NHB | Both | All | NEC | 0.90 |
| Hispanic | Both | All | NEC | NHW | Both | All | NEC | 0.74 |
| NHB | Both | All | NEC | NHW | Both | All | NEC | 0.14 |
| All | Female | All | Adenocarcinoma | All | Male | All | Adenocarcinoma | 0.66 |
| All | Female | All | Adenocarcinoma | All | Both | All | Adenocarcinoma | 0.64 |
| All | Male | All | Adenocarcinoma | All | Both | All | Adenocarcinoma | 0.85 |
| All | Female | All | All | All | Male | All | All | 0.09 |
| All | Female | All | All | All | Both | All | All | 0.07 |
| All | Female | All | GIST | All | Male | All | GIST | 0.84 |
| All | Female | All | GIST | All | Both | All | GIST | 0.92 |
| All | Male | All | GIST | All | Both | All | GIST | 0.94 |
| Hispanic | Female | All | All | Hispanic | Male | All | All | 0.10 |
| Hispanic | Female | All | All | Hispanic | Both | All | All | 0.24 |
| Hispanic | Male | All | All | Hispanic | Both | All | All | 0.05 |
| Hispanic | Female | All | GIST | Hispanic | Male | All | GIST | 0.68 |
| Hispanic | Female | All | GIST | Hispanic | Both | All | GIST | 0.70 |
| Hispanic | Male | All | GIST | Hispanic | Both | All | GIST | 0.72 |
| Hispanic | Female | All | NEC | Hispanic | Male | All | NEC | 0.15 |
| Hispanic | Female | All | NEC | Hispanic | Both | All | NEC | 0.81 |
| All | Male | All | NEC | Hispanic | Both | All | NEC | 0.09 |
| NHB | Female | All | Adenocarcinoma | NHB | Male | All | Adenocarcinoma | 0.93 |
| NHB | Female | All | Adenocarcinoma | NHB | Both | All | Adenocarcinoma | 0.81 |
| NHB | Male | All | Adenocarcinoma | NHB | Both | All | Adenocarcinoma | 0.95 |
| NHB | Female | All | All | NHB | Male | All | All | 0.39 |
| NHB | Female | All | All | NHB | Both | All | All | 0.12 |
| NHB | Male | All | All | NHB | Both | All | All | 0.14 |
| NHB | Female | All | GIST | NHB | Male | All | GIST | 0.26 |
| NHB | Female | All | GIST | NHB | Both | All | GIST | 0.56 |
| NHB | Male | All | GIST | NHB | Both | All | GIST | 0.96 |
| NHB | Female | All | NEC | NHB | Male | All | NEC | 0.17 |
| NHB | Female | All | NEC | NHB | Both | All | NEC | 0.12 |
| NHB | Male | All | NEC | NHB | Both | All | NEC | 0.05 |
| NHW | Female | All | Adenocarcinoma | NHW | male | All | Adenocarcinoma | 0.56 |
| NHW | Female | All | Adenocarcinoma | NHW | Both | All | Adenocarcinoma | 0.28 |
| NHW | Male | All | Adenocarcinoma | NHW | Both | All | Adenocarcinoma | 0.61 |
| NHW | Female | All | All | NHW | Male | All | All | 0.17 |
| NHW | Female | All | All | NHW | Both | All | All | 0.16 |
| NHW | Male | All | All | NHW | Both | All | All | 0.26 |
| NHW | Female | All | GIST | NHB | Male | All | GIST | 0.95 |
| NHW | Female | All | GIST | NHW | Both | All | GIST | 0.93 |
| NHW | Male | All | GIST | NHW | Both | All | GIST | 0.88 |
| NHW | Male | All | NEC | NHW | Both | All | NEC | 0.08 |

Abbreviations: NHW: Non-Hispanic White; NHB: Non-Hispanic Black; NEC: Neuroendocrine carcinoma; GIST: Gastrointestinal stromal tumor.
