# Supplementary material for: A population-based study on incidence trends of small intestine cancer in the United States from 2000 to 2020
Source: PLoS One. 2024 Aug 19;19(8):e0307019. doi: 10.1371/journal.pone.0307019 (PMC11332941; doi:10.1371/journal.pone.0307019)
Supplement: S3 Table — (DOCX) [file pone.0307019.s003.docx]

**S3 Table.** Identical trends of small intestine cancer incidence rate over 2000-2019 in the United States.

| **Race** | **Sex** | **Age** | **Subtype** | **Race** | **Sex** | **Age** | **Subtype** | **P Value** |
| --- | --- | --- | --- | --- | --- | --- | --- | --- |
| **Cohort 1** | | | | **Cohort 2** | | | |  |
| Hispanic | Female | All | Adenocarcinoma | NHW | Female | All | Adenocarcinoma | 0.08 |
| All | Female | All | All | NHW | Female | All | All | 0.50 |
| All | Female | All | GIST | Hispanic | Female | All | GIST | 0.65 |
| All | Female | All | GIST | NHW | Female | All | GIST | 0.27 |
| Hispanic | Female | All | GIST | NHB | Female | All | GIST | 0.06 |
| Hispanic | Female | All | GIST | NHW | Female | All | GIST | 0.14 |
| All | Male | All | GIST | NHB | Male | All | GIST | 0.57 |
| All | Male | All | GIST | NHW | Male | All | GIST | 0.70 |
| Hispanic | Male | All | GIST | NHB | Male | All | GIST | 0.67 |
| Hispanic | Male | All | GIST | NHW | Male | All | GIST | 0.06 |
| NHB | Male | All | GIST | NHW | Male | All | GIST | 0.59 |
| All | Both | All | GIST | NHW | Both | All | GIST | 0.25 |
| Hispanic | Both | All | GIST | NHB | Both | All | GIST | 0.28 |
| NHB | Female | All | GIST | NHB | Both | All | GIST | 0.13 |

Abbreviations: NHW: Non-Hispanic White; NHB: Non-Hispanic Black; GIST: Gastrointestinal stromal tumor.
