# Supplementary material for: A population-based study on incidence trends of small intestine cancer in the United States from 2000 to 2020
Source: PLoS One. 2024 Aug 19;19(8):e0307019. doi: 10.1371/journal.pone.0307019 (PMC11332941; doi:10.1371/journal.pone.0307019)
Supplement: S4 Fig — (DOCX) [file pone.0307019.s007.docx]

 **S4 Fig.** Incident numbers and incidence rate (per 100,000 population) of adenocarcinoma in the United States among males and females in each age group.
