# Supplementary material for: A population-based study on incidence trends of small intestine cancer in the United States from 2000 to 2020
Source: PLoS One. 2024 Aug 19;19(8):e0307019. doi: 10.1371/journal.pone.0307019 (PMC11332941; doi:10.1371/journal.pone.0307019)
Supplement: S11 Fig — APC: annual percent change. * Represent p-value less than 0.05. (DOCX) [file pone.0307019.s014.docx]

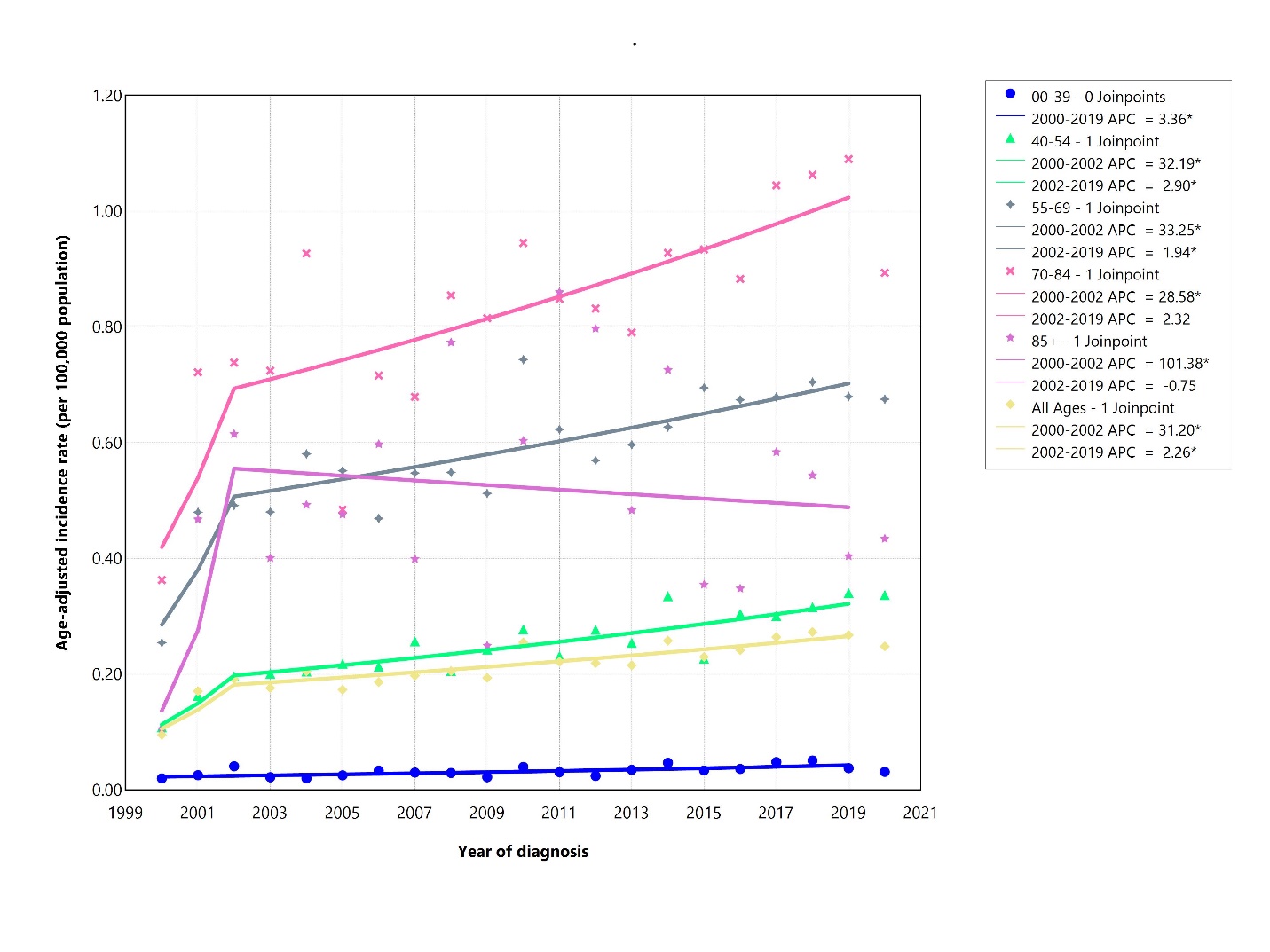


**S11 Fig.** Age-adjusted incidence rate of gastrointestinal stromal tumor over 2000-2019 and in 2020 in the United States, by age. APC: annual percent change. * Represent p-value less than 0.05.
