# Supplementary figures and images for: A population-based study on incidence trends of small intestine cancer in the United States from 2000 to 2020
Source: PLoS One. 2024 Aug 19;19(8):e0307019. doi: 10.1371/journal.pone.0307019 (PMC11332941; doi:10.1371/journal.pone.0307019)

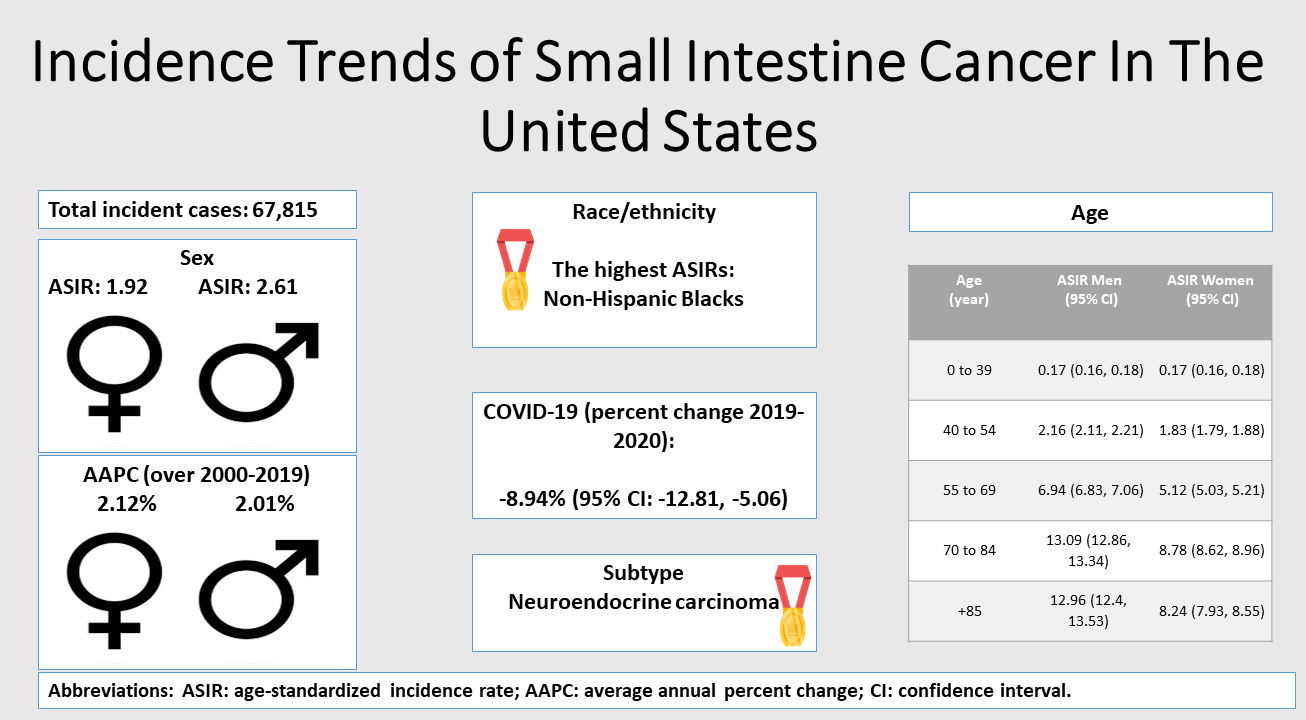

Supplement: S1 Graphical abstract — (TIF) [file pone.0307019.s016.tif]
